# Volatile organic compounds exposure associated with sarcopenia in US adults from NHANES 2011–2018

**DOI:** 10.3389/fpubh.2025.1613435

**Published:** 2025-07-15

**Authors:** Pangbo Wang, Wei Chen, Hongwei Fang, Liwei Xu, Jun Zhao, Jing Huang

**Affiliations:** 1 Hand and Foot Microsurgery, NO. 946 Hospital of PLA Land Force, Yining, Xinjiang, China; 2 Department of Laboratory Medicine, NO. 946 Hospital of PLA Land Force, Yining, Xinjiang, China; 3 Trauma Neurosurgery, NO. 946 Hospital of PLA Land Force, Yining, Xinjiang, China; 4 Department of Neurosurgery and State Key Laboratory of Trauma, Burn and Combined Injury, Southwest Hospital, Chongqing, China; 5 Chongqing Key Laboratory of Precision Neuromedicine and Neuroregenaration, Third Military Medical University (Army Medical University), Chongqing, China; 6 School of Nursing, Peking University, Beijing, China

**Keywords:** volatile organic compounds, sarcopenia, weighted quantile sum regression, Bayesian Kernel machine regression, network pharmacology analysis

## Abstract

**Background:**

Volatile organic compounds (VOCs) are emerging environmental pollutants linked to various health problems. However, the relationship between exposure to urinary volatile organic compound metabolites (mVOCs) and sarcopenia remains unclear.

**Methods:**

We used data from the National Health and Nutrition Examination Survey (NHANES 2011–2018) to assess the association between mVOCs and sarcopenia through multivariable logistic regression and restricted cubic spline (RCS) regression. We also employed Weighted Quantile Sum (WQS) regression model, a high-dimensional statistical approach used to evaluate the joint effects of multiple exposures, and Bayesian Kernel Machine regression (BKMR) model, a combination of Bayesian and statistical learning methods, to assess the mixture effects of mVOCs on sarcopenia risk. These methods account for non-linearity, collinearity, and dimensionality in exposure data. Mediation analysis was used to identify metabolic, endocrine, and inflammatory mediators in these associations. Subgroup analyses were conducted by gender and age. Network pharmacology analysis was performed to identify potential pathways and targets.

**Results:**

A total of 2,898 participants were included, with 145 (8%) diagnosed with sarcopenia. Logistic regression showed a positive correlation between mVOCs (3,4-MHA, ATCA, CEMA, CYMA, 2HPMA, 3HPMA, MHBMA3, and PGA) and sarcopenia. RCS results confirmed linear dose-response associations (*P* for overall < 0.05, *P* for non-linear ≥0.05). Subgroup analysis indicated stronger associations in older participants. The WQS and BKMR models consistently showed a positive link between VOC exposure and sarcopenia. Mediation analysis identified alkaline phosphatase (ALP), white blood cell count (WBC), systemic immune-inflammation index (SII), and vitamin D as mediators. Network analysis revealed significant enrichment in the endocrine resistance pathway.

**Conclusions:**

Our findings suggest that co-exposure to VOCs is associated with increased sarcopenia risk, potentially through disruption of endocrine and inflammatory pathways, as indicated by elevated alkaline phosphatase (ALP), white blood cell count (WBC), the systemic immune-inflammation index (SII), and reduced vitamin D levels, with enrichment observed in the endocrine resistance signaling pathway.

## 1 Background

Sarcopenia is a geriatric condition characterized by a progressive loss of muscle mass, strength and function, and has been proven to increase the risk of falls, fractures, low quality of life, postoperative complications, a loss of independence (1, 2), cognitive impairment, and mortality in general populations. The prevalence of sarcopenia in older adults (aged >60 years) is estimated to range from 10 to 30%, with higher rates observed in older age groups (3–5). The prevalence of sarcopenia among Asian populations ranges from 6.8 to 25.7%, representing a significant social and economic burden (6–8). Risk factors for sarcopenia include age, physical inactivity, smoking, chronic metabolic diseases, malnutrition, and neuromuscular dysfunction (9, 10). Previous studies have highlighted these factors, with particular emphasis on the role of aging, physical inactivity, and metabolic imbalance in increasing the risk of sarcopenia. There is increasing evidence that environment pollutants may play important roles in the development of sarcopenia (11, 12). However, the specific compounds remain unclear.

Volatile organic compounds (VOCs) are carbon-based compounds with low molecular mass that could evaporate easily at normal environment (13–15). They are widespread in both indoor and outdoor environments and the primary sources include but not limited to industrial emissions, automotive exhaust, cooking fumes, cigarettes, insecticides, furniture and building materials, and personal care products (16–18). This poses a significant threat to human health, as individuals spend the majority of their time indoors. VOCs could be absorbed into the human body not only through inhalation of air, but also via dietary intake and dermal contact. Long-term exposure to VOCs has been linked to several health issues, such as chronic respiratory disease (19), growth and development (16), kidney diseases (15, 20), metabolic diseases (21, 22), and depression (23). Due to the longer biological half-life and greater stability of VOCs in urine compared to blood, VOCs in urine serve as reliable indicators for reacting human exposure to these compounds. Additionally, VOCs in urine accumulate over time and reflect long-term exposure, whereas blood concentrations fluctuate more rapidly, potentially leading to underrepresentation of exposure levels. Urinary mVOCs also provide a non-invasive and convenient sampling method, allowing for repeated collection without the need for invasive procedures. Given these advantages, urinary mVOCs are increasingly recognized as a robust biomarker for assessing human exposure to VOCs, particularly in epidemiological studies investigating chronic health outcomes such as sarcopenia. VOCs encompass a variety of species that frequently exist as mixtures in the natural environment. These mixtures can influence both physiological and pathological processes within the body. Several studies have reported the effects of certain VOCs on sarcopenia. For instance, Eshima et al. (24) have found that lipid hydroperoxides promote sarcopenia through carbonyl stress. However, to date, research on the impact of VOC mixtures on sarcopenia remains limited.

In this study, we employed a combination of strategies including survey-weighted logistic regression, restricted cubic spline (RCS) regression, and weighted quantile sum (WQS) regression models to explore the individual and combined effects of exposure to VOCs on sarcopenia. Bayesian kernel machine regression (BKMR) models was used to further validate the mixed effect of VOCs on sarcopenia. Moreover, mediation analysis and network pharmacological analysis were utilized to investigate the potential mechanism between VOCs exposure and sarcopenia. Our findings might provide a novel insight for the understanding of the impact of individual VOCs and their co-exposure on the occurance of sarcopenia.

## 2 Methods

### 2.1 Study population

**Figure 1:**
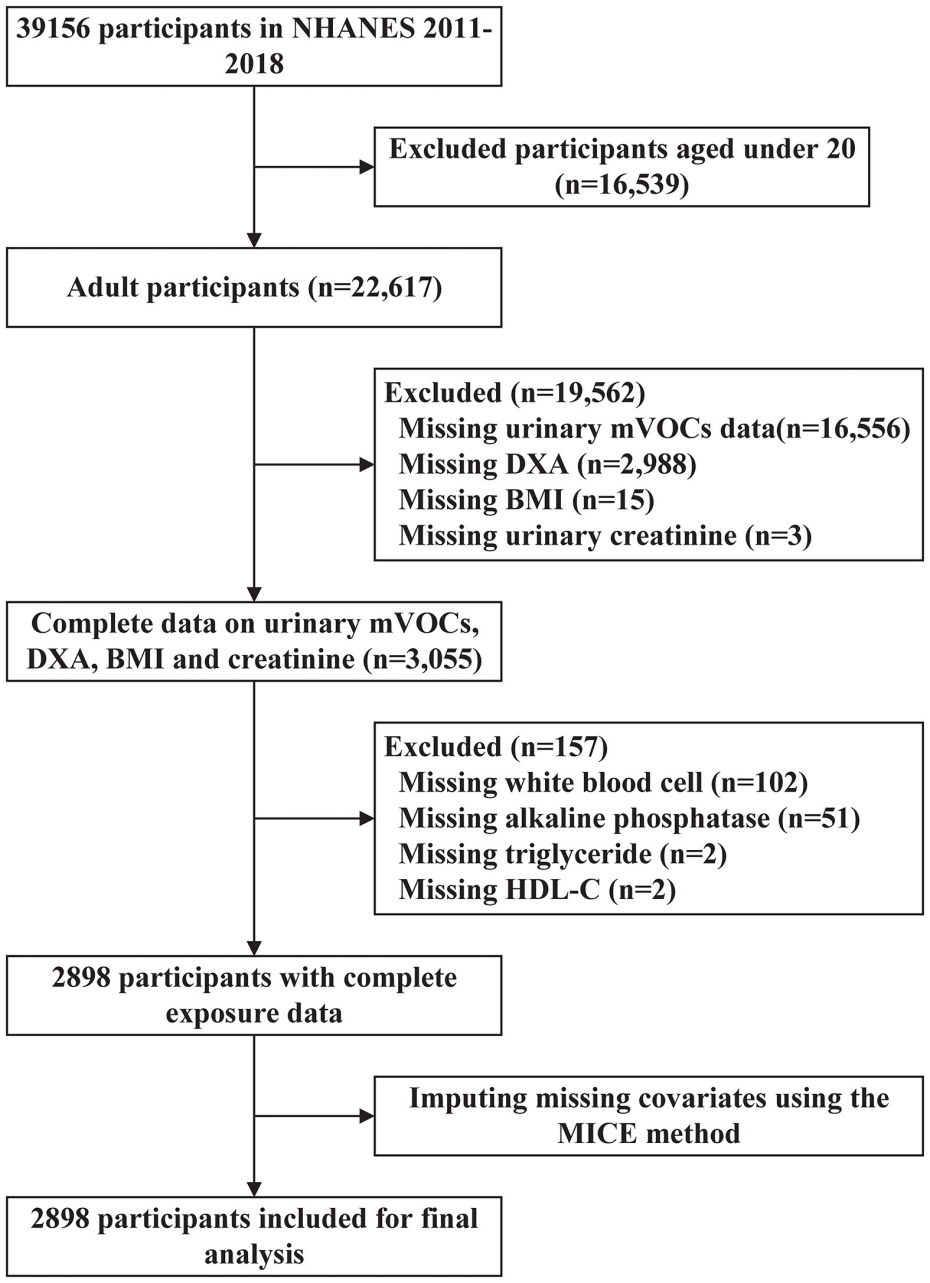
Flowchart of participants selected in this study. NHANES, National Health and Nutrition Examination Survey; mVOCs, volatile organic compound metabolites; DXA, dual-energy X-ray absorptiometry; BMI, body mass index; HDL-C, high-density lipoprotein cholesterol; MICE, multiple imputation methods.

The data used in this cross-sectional study were derived from the 2011–2018 National Health and Nutrition Examination Survey (NHANES), a national, complex, stratified, multistage, probability sampling design survey, conducted by the National Center for Health Statistics (NCHS) of the Centers for Disease Control and Prevention. The purpose of the NHANES was to evaluate the health and nutritional status of the population in the US (25). The NHANES was approved by the NCHS Research Ethics Review Board and all selected participants signed the written informed consent. Among the 39,156 participants, some individuals were excluded based on the following criteria: (1) those who were under 20 years old (*n* = 16,539); (2) those with missing urinary mVOCs data (*n* = 16,556); (3) those with missing data on dual-energy X-ray absorptiometry (DXA) measurements (*n* = 2,988), body mass index (BMI; *n* = 15), and urinary creatinine (*n* = 3); (4) those with missing laboratory test results for white blood cell count (WBC; *n* = 102), alkaline phosphatase (ALP; *n* = 51), triglycerides (*n* = 2), and high-density lipoprotein cholesterol (HDL-C; *n* = 2). Subsequently, covariates with < 20% missing values were imputed using multiple imputation by chained equations (MICE). As shown in Figure 1, a total of 2,898 participants were enrolled in the final analysis.

### 2.2 Determination of urinary VOC metabolites

Urinary volatile organic compound metabolites (mVOCs) were detected and quantified using ultra-performance liquid chromatography-electrospray tandem mass spectrometry (UPLC-ESI/MSMS) as described by Alwis et al. (26). The detailed information on the analytical methods is available on the NHANES website (https://www.cdc.gov/nchs/nhanes/). Based on the NHANES guideline, the concentration of mVOCs was presented as ng/ml. If the concentration of VOCs is below the limit of detection (LOD), the values were replaced by LOD divided by the square root of two. A total of 15 mVOCs were incorporated into the analysis based on their detection rates in the NHANES 2011–2018 dataset, including 2-methylhippuric acid (2MHA), 3- methylhippuric acid & 4- methylhippuric acid (3,4-MHA), N-acetyl-S-(2-carbamoylethyl)-L-cysteine (AAMA), N-Acetyl-S-(N-methylcarbamoyl)-L-cysteine (AMCC), 2-aminothiazoline-4-carboxylic acid (ATCA), N-Acetyl-S-(benzyl)-L-cysteine (SBMA), N-acetyl-S-(2-carboxyethyl)-L-cysteine (CEMA), N-Acetyl-S-(2-cyanoethyl)-L-cysteine (CYMA), N-Acetyl-S-(3,4-dihydroxybutyl)-L-cysteine (DHBMA), N-Acetyl-S-(2-hydroxypropyl)-L-cysteine (2HPMA), N-Acetyl-S-(3-hydroxypropyl)-L-cysteine (3HPMA), N-Acetyl-S-(3-hydroxypropyl-1-methyl)-L-cysteine (HMPMA), Mandelic acid (MA), N-acetyl-S-(4-hydroxy-2-butenyl)-L-cysteine (MHBMA3), and phenylglyoxylic acid (PGA). We chose to include metabolites with a detection rate >80% to ensure that the exposure data was robust and reliable across the study population. Metabolites with a lower detection rate were excluded to avoid introducing significant missing data, which could have compromised the statistical analyses. This threshold is consistent with prior research in environmental health and exposure assessment (16, 27, 28).

### 2.3 Sarcopenia

Sarcopenia was diagnosed based on the sarcopenia index which was defined as the appendicular skeletal muscle mass (ASM), the sum of lean mass for both arms and legs, after adjusting for BMI. The ASM was measured by dual-energy X-ray absorptiometry (DEXA). A sarcopenia index of < 0.512 for females and < 0.789 for males was considered to have sarcopenia (29, 30) according to guidelines from the National Institutes of Health (FNIH).

### 2.4 Covariates

Based on previous studies, the covariates associated with the sarcopenia were identified in the analysis (31, 32). The sociodemographic characteristics included age (years), gender (male and female), race (Mexican American, Other Hispanic, Non-Hispanic White, Non-Hispanic Black, and Other Race), educational level (< 9th grade, 9–11th grade, High school graduate, Some college/AA degree, and College graduate/above), marital status (Married/living with partner, Widowed/separated/divorced, and Never married), and the ratio of family income to poverty (PIR; ≤ 1.0, >1.0 and ≤ 3.0, >3.0). The life behavior characteristics included BMI, smoking status (yes and no), and drinking status (yes and no). The concurrent diseases included hypertension (yes and no), diabetes (yes and no), and stroke (yes and no). The laboratory indicators included white blood cell (WBC), alkaline phosphotase (ALP), platelet, neutrophils, lymphocyte, serum vitamin D, triglycerides, and high-density lipoprotein cholesterol (HDL-C). Family PIR was categorized into 0–1.0, 1.0–3.0, and >3.0. Smokers are defined as individuals who have smoked at least 100 cigarettes in their lifetime and are currently active smokers (33). Alcohol consumers are defined as individuals who consume at least 12 alcohol drinks every year (34).

### 2.5 Statistical analysis

The Baseline features of the participants were described based on the presence or absence of sarcopenia. Continuous variables were presented as mean ± standard deviation (SD) and compared by weighted *t*-test or Kruskal–Wallis test. Categorical variables were expressed as *n* (%) and analyzed using the chi-square test. Due to the right-skewed distribution, the concentration of mVOCs were logarithmically transformed to achieve a normal distribution and grouped into four quartiles (Q1, Q2, Q3, and Q4) for further analysis. Spearman correlation coefficients were calculated to assess the correlations between ln-transformed concentrations of mVOCs. Subgroup analysis was conducted according to gender (male and female group) and age (20 ≤ age ≤ 40 group and age >40 group).

#### 2.5.1 Primary exposure-outcome assessment

The multivariable logistic regression was used to explore the relationship between individual mVOCs and the risk of sarcopenia, with results expressed as odds ratios (ORs) and 95% confidence intervals (CIs). Logistic regression is a robust statistical method that can analyze binary outcomes, such as the presence or absence of sarcopenia, and their associations with both continuous and categorical variables. In this study, mVOCs were analyzed both as continuous and categorical variables, with the first quartile (Q1) serving as the reference group. All models were adjusted for gender, age, race, education, family PIR, marital status, body mass index, smoking status, alcohol drinking, hypertension, diabetes, stroke, white blood cell (WBC), alkaline phosphotase (ALP), platelet, neutrophils, lymphocyte, serum vitamin D, triglycerides, and high-density lipoprotein cholesterol. To minimize potential issues with multicollinearity between the exposure variables and covariates, variance inflation factor (VIF) tests were conducted using SPSS (v 25.0). A VIF value >5 was considered indicative of multicollinearity (35, 36). In this study, WBC, neutrophils, and lymphocyte had VIF values >10, indicating significant multicollinearity. Based on clinical experience, we retained WBC for subsequent analysis and excluded neutrophils and lymphocyte as covariates. In addition, the restricted cubic splines (RCS) with four knots at the 5th, 35th, 65th, and 95th centiles were employed to investigate the dose-response relationship between mVOCs and sarcopenia.

#### 2.5.2 Mixture effects analysis

##### 2.5.2.1 Weighted quantile sum (WQS) regression model

To further assess the joint effects of co-exposure to mVOCs on the risk of sarcopenia, we employed the WQS regression model. Detailed methodological descriptions of WQS regression are available elsewhere (37, 38). Briefly, WQS is a high-dimensional statistical approach that integrates weighted quantiles into either linear regression models (for continuous outcomes) or logistic regression models (for binary outcomes) (38, 39). This method constructs a composite weighted index based on quantiles of each exposure component and evaluates its association with the outcome of interest. It enables the estimation of mixture effects while accounting for the dimensionality of co-exposure, as well as potential non-linearity and collinearity among the exposure variables. In our analysis, the dataset was randomly split into training and validation sets in a 4:6 ratio. Using the training set, we performed 1,000 bootstrap resampling iterations to preliminarily derive the WQS weights for each mVOC. These weights, constrained to range from 0 to 1 and sum to 1 across all components, were then applied to the validation set to assess the statistical significance of the associations. Since WQS regression requires the assumption that all components contribute to the outcome in the same direction, we performed two separate models—one assuming a positive association and the other a negative association between mVOCs and sarcopenia. Additionally, the WQS regression models were adjusted for the same set of covariates as the logistic regression models described earlier, to control for potential confounding factors.

##### 2.5.2.2 Bayesian kernel machine regression (BKMR) model

We employed the BKMR model, a combination of bayesian and statistical learning methods, to explore the joint effects of mVOCs on the risk of sarcopenia. The overall effect of the mVOCs mixture was assessed by comparing the changes in effects between specific quantiles and the median of the mVOCs mixture. Additionally, we investigated the univariate exposure-response function by evaluating the impact of individual mVOCs on sarcopenia risk when the other mVOCs were fixed at their median values, with a particular focus on the effects when an individual mVOC was at the 75th and 25th percentiles. To evaluate the weight index of each mVOC's influence on sarcopenia risk, we used the posterior inclusion probability (PIP), with a PIP threshold of 0.5 defined as statistically significant. The bivariate exposure-response curves were used to demonstrate the interactions between different mVOCs in the mixture. Specifically, the effect of the target mVOC on sarcopenia risk was assessed at the 10th, 50th, and 90th percentiles of another mVOC in the mixture. The regression models were adjusted for the same potential confounding factors mentioned earlier. All analyses were conducted using the Markov Chain Monte Carlo (MCMC) method with 20,000 iterations. Furthermore, based on the Spearman correlations among the mVOCs, the 15 mVOCs were grouped into four clusters (Group 1: 2MHA, 3,4-MHA; Group 2: AAMA, AMCC, CEMA, DHBMA, 2HPMA, MA, PGA; Group 3: ATCA, SBMA; Group 4: CYMA, 3HPMA, MHBMA3, HMPMA) to fit a stratified BKMR model.

#### 2.5.3 Mechanistic investigations

##### 2.5.3.1 Mediation effect analysis

In our study, mediation analysis was applied to explore whether metabolic factors, endocrine factors, and inflammation biomarkers mediate the associations between mVOCs and sarcopenia. The exposure variable was the mVOCs mixture (X), the outcome variable was sarcopenia (Y) and the mediating factors was metabolic factors, endocrine factors, or inflammation biomarkers (M). The total effect (TE) of mVOCs was divided into direct effect (DE) and indirect effect (IE). The direct effect represents the influence of mVOC exposure on sarcopenia without the mediation of other factors, while the indirect effect represents the impact of mVOC exposure on sarcopenia through the mediators. The proportion of the indirect effect in the total effect (IE/TE) indicates the mediating variable's effectiveness, reflecting the extent to which the mediator explains the relationship between mVOC exposure and sarcopenia risk. According to previous research, TG/HDL-C was selected as an indicator of metabolic factors (21), vitamin D as a biomarker of endocrine factors (40) and SII, WBC and ALP as markers of inflammation (41, 42). The formulas for the calculation of TG/HDL-C and SII are as follows TG/HDL-C = triglycerides (mg/dl)/HDL-C (mg/dl) and SII = platelet count × neutrophil count/lymphocyte count.

##### 2.5.3.2 Network pharmacological analysis

The pharmacological targets of VOCs were screened using the Drugbank (https://go.drugbank.com/) and SwissTargetPrediction (http://www.swisstargetprediction.ch/) databases. The protein targets related to sarcopenia were obtained using GeneCards database (https://www.genecards.org/). The primary targets of VOCs and sarcopenia were overlapped and analyzed via Venn diagrams to identify potential targets of VOCs contributing to depression. Subsequently, the STRING database (https://cn.string-db.org/) was utilized to construct a protein-protein interaction (PPI) network among the intersecting targets, with the confidence threshold for PPI analysis set to >0.4. With the help of the Analyze Network tool, core targets were identified based on parameters such as maximal clique centrality (MCC) and degree of network nodes. Using the Database for Annotation, Visualization, and Integrated Discovery (DAVID; https://davidbioinformatics.nih.gov/), Kyoto Encyclopedia of Genes and Genomes (KEGG) and Gene Ontology (GO) pathway enrichment analysis were performed on the core targets of VOCs and sarcopenia to identify potential interaction pathways.

Although the sample size for sarcopenia patients (*n* = 145) is relatively small, we adhered to the event-to-variable ratio (EVR) principle, ensuring that the number of covariates did not exceed the recommended 10 events per variable. To mitigate overfitting, we carefully selected covariates and applied various models (e.g., WQS and BKMR) for cross-validation. Furthermore, we performed VIF analysis to detect multicollinearity and excluded variables with high collinearity, such as neutrophils and lymphocytes. We believe that, despite the smaller sample size, the study design and analytical methods adequately support our conclusions.

All regression models were adjusted for the same covariates as in the logistic regression. All analyses were performed with R software (version 4.4.1). The “corrplot” (version 0.94), “plotRCS” (version 0.1.4), “gWQS” (version 3.0.5), “bkmr” (version 0.2.2), “mediation” (version 4.5.0) and “glmnet” (version 4.1-8) packages were utilized for correlation analysis, RCS models, WQS regression, BKMR regression, and mediation analysis, respectively. The statistical significance was defined as *P* value of < 0.05 (two-sided).

## 3 Results

### 3.1 Population characteristics

A total of 2,898 participants were eventually included in the analysis from NHANES 2011–2018. The demographic characteristics were presented in Table 1. Among them, 145 (8%) cases were diagnosed with sarcopenia. Compared with participants without sarcopenia, those with sarcopenia were more likely to be older, female, Mexican American, and to experience family discord, lower education levels, lower family incomes, higher BMI, a lower prevalence of smoking and drinking, and a higher prevalence of hypertension and diabetes (all *P* < 0.05). There were also significant differences between the two groups in terms of WBC, ALP, and platelet (all *P* < 0.05). The correlation between 15 ln-transformed mVOCs is presented in Supplementary Figure S1 using Spearman correlation coefficients. Three mVOCs (MEOHP, MEHHP, and MECPP) were strongly correlated with each other, with correlation coefficients >0.7. Additionally, there were strong correlations between MCPP and MCOP (*r* = 0.73).

**Table 1:** Baseline characteristics of included participants.

**Variables**	**Overall (*n* = 2,898)**	**Non-sarcopenia (*n* = 2,753)**	**Sarcopenia (*n* = 145)**	***P*-value**
Age, years (mean ± SD)	39.14 ± 11.5	38.86 ± 11.39	44.59 ± 11.64	**0.000**
**Gender**, ***n*** **(%)**				**0.000**
Male	1,450 (50.0)	1,425 (51.8)	25 (17.2)	
Female	1,448 (50.0)	1,328 (48.2)	120 (82.8)	
**Race**, ***n*** **(%)**				**0.000**
Mexican American	414 (14.3)	367 (13.3)	47 (32.4)	
Other Hispanic	328 (11.3)	298 (10.8)	30 (20.7)	
Non-Hispanic White	975 (33.6)	941 (34.2)	34 (23.4)	
Non-Hispanic Black	617 (21.3)	606 (22.0)	11 (7.6)	
Other Race	564 (19.5)	541 (19.7)	23 (15.9)	
**Education**, ***n*** **(%)**				**0.000**
< 9th grade	176 (6.1)	153 (5.6)	23 (15.9)	
9–11th grade	337 (11.6)	319 (11.6)	18 (12.4)	
High school graduate	634 (21.9)	594 (21.6)	40 (27.6)	
Some college/AA degree	924 (31.9)	883 (32.1)	41 (28.3)	
College graduate/above	827 (28.5)	804 (29.2)	23 (15.9)	
**Marital status**, ***n*** **(%)**				**0.035**
Married/living with partner	1,722 (59.4)	1,628 (59.1)	94 (64.8)	
Widowed/separated/divorced	379 (13.1)	351 (12.7)	28 (19.3)	
Never married	797 (27.5)	774 (28.1)	23 (15.9)	
**Family PIR**, ***n*** **(%)**				**0.038**
≤ 1.0	612 (21.1)	578 (21.0)	34 (23.4)	
>1.0, ≤ 3.0	1,168 (40.3)	1,099 (39.9)	69 (47.6)	
>3.0	1,118 (38.6)	1,076 (39.1)	42 (29.0)	
BMI, kg/m^2^ (mean ± SD)	28.80 ± 6.79	28.50 ± 6.58	34.49 ± 8.07	**0.000**
**Smoke**, ***n*** **(%)**				**0.014**
Yes	638 (22.0)	618 (22.4)	20 (13.8)	
No	2,260 (78.0)	2,135 (77.6)	125 (86.2)	
**Alcohol drinking**, ***n*** **(%)**				**0.000**
Yes	2,297 (79.3)	2,199 (79.9)	98 (67.6)	
No	601 (20.7)	554 (20.1)	47 (32.4)	
**Hypertension**, ***n*** **(%)**				**0.000**
Yes	2,156 (74.4)	2,028 (73.7)	128 (88.3)	
No	742 (25.6)	725 (26.3)	17 (11.7)	
**Diabetes**, ***n*** **(%)**				**0.000**
Yes	273 (9.4)	240 (8.7)	33 (22.8)	
No	2,625 (90.6)	2,513 (91.3)	112 (77.2)	
**Stroke**, ***n*** **(%)**				0.522
Yes	42 (1.4)	39 (1.4)	3 (2.1)	
No	2,856 (98.6)	2,714 (98.6)	142 (97.9)	
WBC, 1,000 cells/μl (mean ± SD)	7.29 ± 2.12	7.25 ± 2.11	8.08 ± 2.25	**0.000**
Alkaline phosphotase, μ/L (mean ± SD)	68.34 ± 22.58	67.88 ± 22.56	77.02 ± 21.23	**0.000**
Platelet, 1,000 cells/μl (mean ± SD)	244.30 ± 59.40	243.08 ± 58.83	267.40 ± 65.46	**0.000**
Serum vitamin D, nmol/L (mean ± SD)	60.12 ± 25.15	60.18 ± 25.19	59.07 ± 24.55	0.638
Triglycerides, mg/dl (mean ± SD)	147.22 ± 118.08	147.17 ± 119.74	148.24 ± 80.41	0.879
HDL, mg/dl (mean ± SD)	52.14 ± 15.03	52.17 ± 15.16	51.70 ± 12.29	0.663

The continuous variables were presented as median (interquartile range, IQR), and the categorical variables were shown as number and percentages. BMI, body mass index; Family PIR, ratio of family income to poverty; SD, standard deviation; vitamin D, 25OHD2 + 25OHD3; WBC, white blood cell; HDL, high-density lipoprotein cholesterol. The *P*-value in bold indicates a statistical significance.

### 3.2 Association between mVOCs exposure and sarcopenia using logistic regression model

Multivariate logistic regression model was utilized to evaluate the association between mVOCs and the risk of sarcopenia. After adjusting for multiple potential confounding factors described above, continuous analysis showed a positive association between the risk of sarcopenia and ln-transformed 3,4-MHA (OR: 1.32, 95% CI: 1.10–1.59), AMCC (OR: 1.50, 95% CI: 1.11–2.02), ATCA (OR: 1.47, 95% CI: 1.14–1.90), CEMA (OR: 1.64, 95% CI: 1.22–2.19), CYMA (OR: 1.17, 95% CI:1.01–1.36), 3HPMA (OR: 1.45, 95% CI: 1.12–1.88), PGA (OR: 1.54, 95% CI: 1.02–2.33), and HMPMA (OR: 1.36, 95% CI: 1.03–1.79). When the mVOCs were analyzed as categorical variables, compared with the control group (Q1), the adjusted logistic model revealed that 3,4-MHA (Q4), ATCA (Q4), CEMA (Q3, Q4), CYMA (Q4), 2HPMA (Q4), 3HPMA (Q3, Q4), MHBMA3 (Q4), and PGA (Q4) were positively correlated with the risk of sarcopenia (Table 2 and Supplementary Tables S1–S30).

**Table 2:** Association of urine mVOCs with sarcopenia in all participants and their subgroup after adjusting for all covariates.

**mVOCs**	**Odds ratios (95% CI)**					***P* for trend**
	**Continuous**	**Q1**	**Q2**	**Q3**	**Q4**	
**2MHA**						
Overall	1.19 (0.99–1.43)	Ref	0.74 (0.43–1.28)	1.30 (0.78–2.17)	1.58 (0.90–2.78)	0.071
Male	0.90 (0.58–1.38)	Ref	0.41 (0.11–1.51)	0.92 (0.28–3.06)	0.65 (0.17–2.54)	0.555
Female	1.28 (1.04–1.58)	Ref	0.84 (0.45–1.56)	1.64 (0.92–2.93)	**2.11 (1.11**–4.04)^*^	0.024
Age (20–40)	0.91 (0.67–1.24)	Ref	0.62 (0.28–1.39)	0.71 (0.30–1.66)	0.72 (0.27–1.88)	0.671
Age (>40)	**1.37 (1.09–1.73)** ^*^	Ref	0.81 (0.37–1.76)	2.39 (1.19–4.79)	**2.45 (1.15**–**5.25)**	0.005
**3,4-MHA**						
Overall	**1.32 (1.10–1.59)** ^*^	Ref	1.39 (0.80–2.41)	1.67 (0.95–2.96)	**2.64 (1.46**–4.74)^*^	0.012
Male	1.17 (0.74–1.87)	Ref	1.41 (0.40–4.98)	1.95 (0.51–7.48)	1.78 (0.40–7.93)	0.792
Female	**1.37 (1.11–1.69)** ^*^	Ref	1.29 (0.69–2.41)	**2.00 (1.08**–3.72)^*^	**3.07 (1.59**–5.96)^*^	0.005
Age (20–40)	1.08 (0.80–1.46)	Ref	0.82 (0.36–1.90)	0.86 (0.33–2.21)	1.49 (0.62–3.58)	0.574
Age (>40)	**1.46 (1.14–1.86)** ^*^	Ref	1.36 (0.63–2.92)	**2.89 (1.36**–6.14)^*^	**3.43 (1.52**–7.75)^*^	0.005
**AAMA**						
Overall	1.11 (0.83–1.48)	Ref	1.21 (0.71–2.05)	1.29 (0.76–2.18)	1.08 (0.57–2.02)	0.787
Male	**2.10 (1.08**–4.09)^*^	Ref	1.13 (0.30–4.26)	2.15 (0.60–7.76)	2.65 (0.61–11.63)	0.435
Female	0.92 (0.67–1.27)	Ref	1.49 (0.82–2.68)	1.24 (0.68–2.25)	0.94 (0.47–1.89)	0.440
Age (20–40)	1.10 (0.71–1.72)	Ref	0.92 (0.39–2.16)	1.09 (0.46–2.61)	1.17 (0.44–3.07)	0.963
Age (>40)	1.12 (0.75–1.66)	Ref	1.45 (0.71–2.93)	1.42 (0.71–2.86)	0.99 (0.42–2.34)	0.584
**AMCC**						
Overall	**1.50 (1.11–2.02)** ^*^	Ref	1.09 (0.59–2.03)	1.76 (0.98–3.18)	1.93 (0.99–3.78)	0.086
Male	**2.65 (1.24–5.66)** ^*^	Ref	2.21 (0.38–12.77)	**5.38 (1.04–27.53)** ^*^	**7.91 (1.16**–53.87)^*^	0.100
Female	1.32 (0.94–1.84)	Ref	1.19 (0.63–2.26)	1.52 (0.80–2.86)	1.65 (0.78–3.51)	0.494
Age (20–40)	1.43 (0.89–2.32)	Ref	0.44 (0.16–1.23)	1.38 (0.61–3.15)	1.59 (0.57–4.46)	0.103
Age (>40)	**1.70 (1.13–2.56)** ^*^	Ref	**3.49 (1.43**–8.55)^*^	**3.13 (1.23**–7.97)^*^	**4.69 (1.65**–13.30)^*^	0.024
**ATCA**						
Overall	**1.47 (1.14–1.90)** ^*^	Ref	1.41 (0.67–2.99)	1.06 (0.50–2.26)	**2.62 (1.28**–5.38)^*^	**0.001**
Male	1.20 (0.73–1.95)	Ref	0.78 (0.19–3.13)	1.37 (0.39–4.84)	1.11 (0.32–3.90)	0.867
Female	**1.62 (1.19–2.20)**	Ref	1.07 (0.53–2.18)	1.76 (0.92–3.36)	**2.39 (1.25**–4.58)^*^	0.018
Age (20–40)	1.33 (0.86–2.06)	Ref	1.16 (0.31–4.36)	1.40 (0.39–5.04)	2.11 (0.61–7.33)	0.450
Age (>40)	**1.66 (1.19–2.31)** ^*^	Ref	1.23 (0.48–3.13)	0.97 (0.38–2.48)	**3.26 (1.32**–8.03)^*^	**0.001**
**SBMA**						
Overall	0.98 (0.79–1.22)	Ref	0.81 (0.47–1.38)	0.73 (0.42–1.26)	0.82 (0.48–1.38)	0.720
Male	1.12 (0.68–1.84)	Ref	0.69 (0.19–2.56)	1.06 (0.32–3.54)	0.80 (0.23–2.83)	0.907
Female	0.94 (0.74–1.20)	Ref	1.04 (0.58–1.87)	0.88 (0.48–1.61)	1.09 (0.61–1.95)	0.905
Age (20–40)	1.02 (0.73–1.43)	Ref	0.64 (0.24–1.71)	0.92 (0.38–2.21)	1.14 (0.49–2.68)	0.659
Age (>40)	0.96 (0.71–1.30)	Ref	0.89 (0.45–1.78)	0.80 (0.39–1.66)	0.85 (0.42–1.71)	0.940
**CEMA**						
Overall	**1.64 (1.22–2.19)** ^*^	Ref	1.49 (0.82–2.70)	**2.66 (1.50–4.72)** ^*^	**2.75 (1.48**–5.10)^*^	**0.002**
Male	1.89 (0.88–4.03)	Ref	0.80 (0.21–3.10)	1.40 (0.37–5.33)	2.94 (0.77–11.19)	0.235
Female	**1.58 (1.14–2.18)** ^*^	Ref	1.41 (0.71–2.82)	**3.50 (1.82–6.73)** ^*^	**2.45 (1.18**–5.09)^*^	**0.000**
Age (20–40)	**1.77 (1.12–2.78)** ^*^	Ref	1.61 (0.64–4.03)	2.01 (0.81–4.98)	**2.80 (1.08**–7.28)^*^	0.197
Age (>40)	**1.66 (1.10–2.50)** ^*^	Ref	1.20 (0.55–2.64)	**2.92 (1.41**–6.06)^*^	1.98 (0.85–4.62)	**0.013**
**CYMA**						
Overall	**1.17 (1.01–1.36)** ^*^	Ref	1.41 (0.85–2.34)	1.48 (0.87–2.52)	**2.77 (1.25**–6.15)^*^	0.086
Male	**1.48 (1.11–1.99)** ^*^	Ref	1.59 (0.48–5.25)	1.23 (0.32–4.82)	**7.21 (1.28**–40.68)^*^	0.140
Female	1.09 (0.91–1.31)	Ref	1.39 (0.78–2.48)	1.69 (0.94–3.05)	1.65 (0.69–3.97)	0.352
Age (20–40)	**1.34 (1.06–1.69)** ^*^	Ref	0.81 (0.34–1.94)	1.47 (0.62–3.49)	**5.02 (1.56–16.13)** ^*^	0.020
Age (>40)	1.08 (0.87–1.34)	Ref	**2.01 (1.01**–4.01)^*^	1.76 (0.87–3.55)	2.14 (0.66–6.89)	0.212
**DHBMA**						
Overall	1.65 (0.98–2.77)	Ref	1.62 (0.84–3.09)	1.96 (1.03–3.71)	1.78 (0.92–3.47)	0.228
Male	2.33 (0.66–8.18)	Ref	0.38 (0.06–2.33)	2.08 (0.56–7.76)	2.27 (0.57–9.03)	0.129
Female	1.51 (0.84–2.71)	Ref	1.76 (0.91–3.40)	1.69 (0.87–3.28)	1.26 (0.61–2.58)	0.272
Age (20–40)	2.09 (0.88–4.99)	Ref	0.96 (0.31–2.96)	**3.56 (1.38**–9.17)^*^	1.57 (0.54–4.60)	0.005
Age (>40)	1.61 (0.83–3.15)	Ref	0.72 (0.32–1.62)	1.38 (0.65–2.95)	1.09 (0.49–2.41)	0.320
**2HPMA**						
Overall	1.19 (0.98–1.45)	Ref	1.21 (0.71–2.05)	1.34 (0.77–2.34)	**1.76 (1.00–3.10)** ^*^	0.253
Male	**1.62 (1.10–2.40)** ^*^	Ref	1.12 (0.28–4.56)	2.31 (0.62–8.64)	3.44 (0.92–12.90)	0.211
Female	1.09 (0.86–1.38)	Ref	1.47 (0.82–2.65)	1.40 (0.75–2.61)	1.82 (0.94–3.52)	0.342
Age (20–40)	1.18 (0.89–1.57)	Ref	0.31 (0.11–0.90)	1.01 (0.44–2.31)	1.44 (0.62–3.34)	**0.039**
Age (>40)	1.19 (0.89–1.59)	Ref	**2.28 (1.13**–4.62)^*^	1.79 (0.82–3.91)	2.10 (0.93–4.76)	0.134
**3HPMA**						
Overall	**1.45 (1.12–1.88)** ^*^	Ref	1.29 (0.73–2.27)	**2.03 (1.18**–3.48)^*^	**2.13 (1.12**–4.04)^*^	0.034
Male	1.91 (0.99–3.71)	Ref	1.82 (0.40–8.25)	5.18 (1.21–22.19)	4.14 (0.72–23.75)	0.110
Female	**1.37 (1.03–1.82)** ^*^	Ref	0.98 (0.52–1.84)	1.67 (0.91–3.05)	1.91 (0.94–3.88)	0.108
Age (20–40)	**1.90 (1.28–2.81)** ^*^	Ref	0.92 (0.35–2.44)	2.06 (0.86–4.92)	**2.98 (1.13**–7.91)^*^	0.057
Age (>40)	1.28 (0.88–1.85)	Ref	0.97 (0.47–2.01)	1.75 (0.88–3.49)	1.52 (0.60–3.84)	0.276
**MA**						
Overall	1.26 (0.89–1.79)	Ref	1.05 (0.61–1.82)	1.42 (0.84–2.41)	1.30 (0.71–2.38)	0.527
Male	1.01 (0.41–2.49)	Ref	1.30 (0.34–4.96)	0.66 (0.15–3.03)	2.20 (0.50–9.64)	0.399
Female	1.29 (0.87–1.90)	Ref	1.11 (0.62–2.00)	1.29 (0.72–2.32)	1.17 (0.60–2.28)	0.862
Age (20–40)	1.64 (0.91–2.97)	Ref	0.89 (0.36–2.20)	1.06 (0.43–2.59)	1.66 (0.64–4.34)	0.590
Age (>40)	1.14 (0.72–1.80)	Ref	0.99 (0.48–2.06)	1.67 (0.84–3.32)	1.18 (0.51–2.74)	0.360
**MHBMA3**						
Overall	1.28 (0.99–1.65)	Ref	1.32 (0.78–2.23)	1.19 (0.68–2.09)	**2.83 (1.47**–5.47)^*^	**0.012**
Male	1.43 (0.78–2.62)	Ref	0.81 (0.23–2.86)	1.02 (0.29–3.68)	3.61 (0.84–15.62)	0.201
Female	1.25 (0.94–1.66)	Ref	1.21 (0.68–2.18)	1.20 (0.64–2.23)	**2.08 (1.01**–4.28)^*^	0.240
Age (20–40)	**1.71 (1.14–2.55)** ^*^	Ref	0.79 (0.31–1.99)	1.44 (0.58–3.59)	**5.01 (1.92–13.06)** ^*^	**0.001**
Age (>40)	1.15 (0.81–1.62)	Ref	1.40 (0.71–2.75)	1.15 (0.56–2.36)	1.85 (0.70–4.84)	0.561
**PGA**						
Overall	**1.54 (1.02–2.33)** ^*^	Ref	1.31 (0.72–2.39)	**1.82 (1.01–3.30)** ^*^	1.93 (0.99–3.74)	0.141
Male	2.08 (0.85–5.09)	Ref	1.12 (0.29–4.33)	1.57 (0.40–6.25)	2.95 (0.72–12.07)	0.430
Female	1.41 (0.87–2.30)	Ref	1.11 (0.60–2.05)	1.42 (0.78–2.60)	1.69 (0.86–3.34)	0.416
Age (20–40)	**2.30 (1.20–4.39)** ^*^	Ref	3.01 (0.96–9.44)	**5.06 (1.68–15.22)** ^*^	**5.03 (1.49–16.95)** ^*^	**0.028**
Age (>40)	1.30 (0.76–2.24)	Ref	0.86 (0.41–1.81)	1.43 (0.72–2.86)	1.52 (0.67–3.46)	0.374
**HMPMA**						
Overall	**1.36 (1.03–1.79)** ^*^	Ref	1.29 (0.73–2.29)	1.58 (0.90–2.76)	2.01 (0.99–4.07)	0.236
Male	**1.99 (1.00–3.99)** ^*^	Ref	0.61 (0.14–2.61)	2.08 (0.60–7.27)	2.65 (0.50–14.03)	0.235
Female	1.26 (0.93–1.72)	Ref	1.64 (0.89–3.03)	1.30 (0.69–2.46)	1.72 (0.78–3.78)	0.373
Age (20–40)	**1.85 (1.23–2.80)** ^*^	Ref	1.62 (0.58–4.51)	**3.33 (1.27**–8.68)^*^	**4.56 (1.53–13.62)** ^*^	0.020
Age (>40)	1.17 (0.79–1.72)	Ref	1.08 (0.54–2.17)	0.96 (0.46–2.01)	1.69 (0.63–4.57)	0.657

^*^P value < 0.05.

Models were adjusted for gender, age, race, education, family PIR, marital status, body mass index, smoking status, alcohol drinking, hypertension, diabetes, stroke, white blood cell, alkaline phosphotase, platelet, serum vitamin D, triglycerides, and high-density lipoprotein cholesterol.

Continuous, ln-transformed concentration of variables; CI, confidence interval; Q, quartile; mVOCs, metabolites of volatile organic compounds.

The bold values indicate statistical significance.

In the age-stratified subgroup analysis, 2MHA (OR: 1.37, 95% CI: 1.09–1.73), 3,4-MHA (OR: 1.46, 95% CI: 1.14–1.86), AMCC (OR: 1.70, 95% CI: 1.13–2.56), ATCA (OR: 1.66, 95% CI: 1.19–2.31), and CEMA (OR: 1.66, 95% CI: 1.10–2.50) remained positively associated with sarcopenia as continuous variables in the age >40 group. CEMA (OR: 1.77, 95% CI: 1.12–2.78), CYMA (OR: 1.34, 95% CI: 1.06–1.69), 3HPMA (OR:1.90, 95% CI: 1.28–2.81), MHBMA3 (OR: 1.71, 95% CI: 1.14–2.55), PGA (OR: 2.30, 95% CI: 1.20–4.39), and HMPMA (OR: 1.85, 95% CI: 1.23–2.80) were associated with sarcopenia as continuous variables in the 20 ≤ age ≤ 40 group. This positive association predominantly persisted when mVOCs were treated as categorical variables. In the gender-stratified subgroup analysis, when mVOCs were considered as continuous variables, AMCC (OR: 2.65, 95% CI: 1.24–5.66), CYMA (OR: 1.48, 95% CI: 1.11–1.99), 2HPMA (OR: 1.62, 95% CI: 1.10–2.40), and HMPMA (OR: 1.99, 95% CI: 1.00–3.99) were closely positively associated with the risk of sarcopenia in the male group, while 3,4-MHA (OR: 1.37, 95% CI: 1.11–1.69), ATCA (OR: 1.62, 95% CI: 1.19–2.20), CEMA (OR: 1.58, 95% CI: 1.14–2.18), and 3HPMA (OR: 1.37, 95% CI: 1.03–1.82) were positively correlated with the risk of sarcopenia in the same group. A similar trend persisted when these variables were considered as categorical variables.

The adjusted RCS regression model was utilized to explore the dose-response relationship between mVOCs and sarcopenia (Supplementary Figure S2). The RCS results further validated linear dose-response associations between the sarcopenia and the mVOCs 2MHA, AMCC, ATCA, CEMA, 3HPMA, and PGA (all *P* for overall < 0.05, all *P* for non-linear ≥0.05). However, no statistically significant non-linear relationship has been found between the mVOCs and the risk of sarcopenia (all *P* for non-linear ≥0.05).

### 3.3 Weighted quantile sum (WQS) regression to assess the associations of mVOCs co-exposure and sarcopenia risk

**Figure 2:**
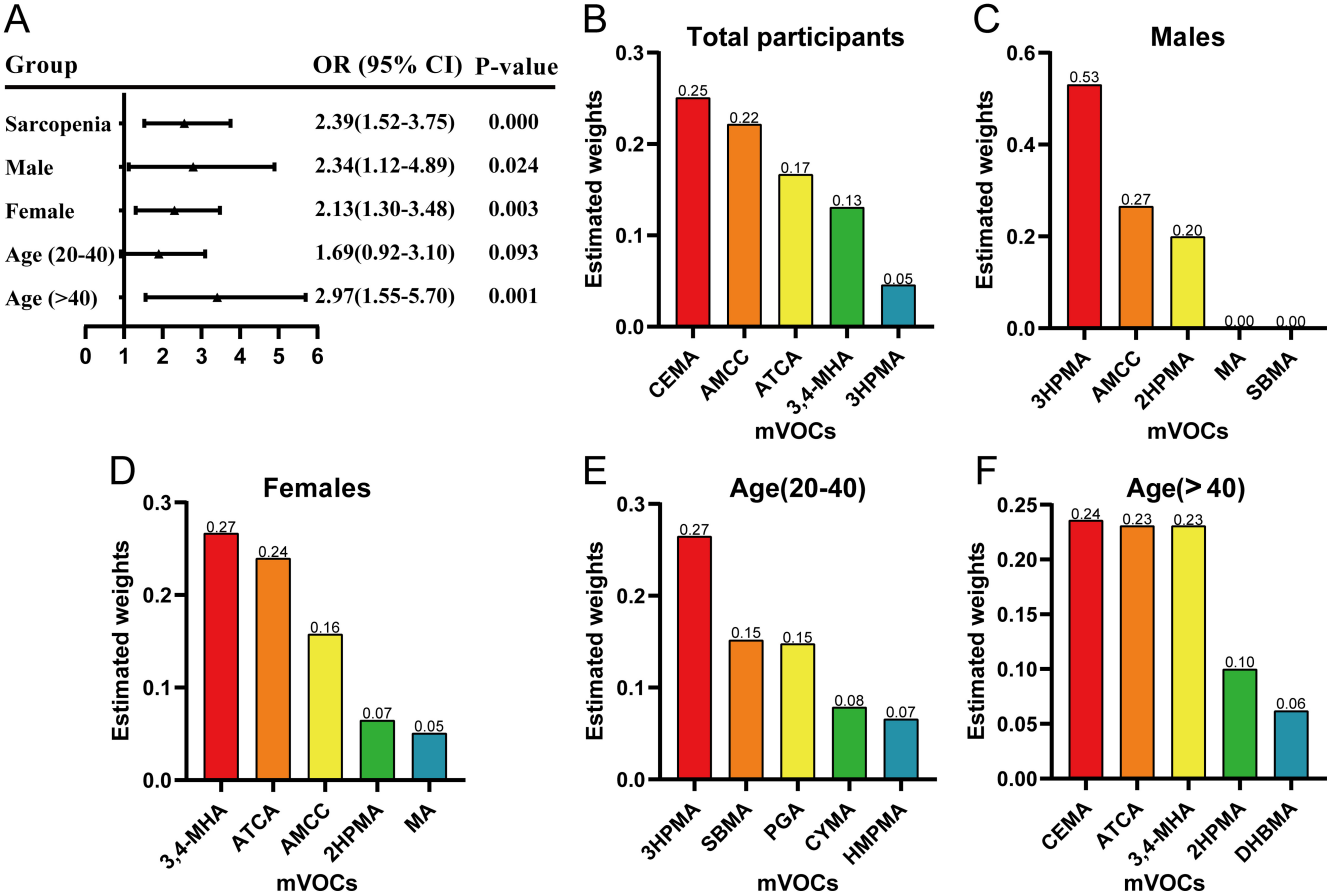
The combined effect of urinary volatile organic compound metabolites (mVOCs) on sarcopenia estimated by the weighted quantile sum (WQS) models in total population and their subgroups. **(A)** The association of mVOCs co-exposure with the risk of sarcopenia in total participants and subgroups stratified by age and gender. The proportional contribution of each mVOC to the combined effect on sarcopenia in all participants **(B)**, males **(C)**, females **(D)**, 20 < age ≤ 40 years **(E)**, and age >40 years **(F)**. The figure illustrates the top five mVOCs ranked by weight. The WQS regression model was adjusted for gender, age, race, education, family PIR, marital status, body mass index, smoking status, alcohol drinking, hypertension, diabetes, stroke, white blood cell, ALP, platelet, serum vitamin D, triglycerides, and high-density lipoprotein cholesterol.

WQS model was used to investigate the mixed effects of 15 mVOCs and the risk of sarcopenia. As shown in Figure 2, the WQS index of mVOCs co-exposure was positively associated with the prevalence of sarcopenia after adjusting for the multivate covariates as in the logistic regression (OR: 2.39, 95% CI: 1.52–3.75, *P* = 0.00). CEMA had the highest weight (25%) among the mVOCs in the positive direction, followed by AMCC (22%), ATCA (17%), 3,4-MHA (13%), and 3HPMA (5%). In the subgroups of males (OR: 2.34, 95% CI: 1.12–4.89, *P* = 0.024), females (OR: 2.13, 95% CI: 1.30–3.38, *P* = 0.003), and individuals aged >40 (OR: 2.97, 95% CI: 1.55–5.70, *P* = 0.001), exposure to combined mVOCs also exhibited a positive trend with regard to sarcopenia. The proportional weight of each mVOC varied across the subgroups.

### 3.4 Bayesian kernel machine regression analysis (BKMR) to assess the associations of mVOCs co-exposure and sarcopenia risk

**Figure 3:**
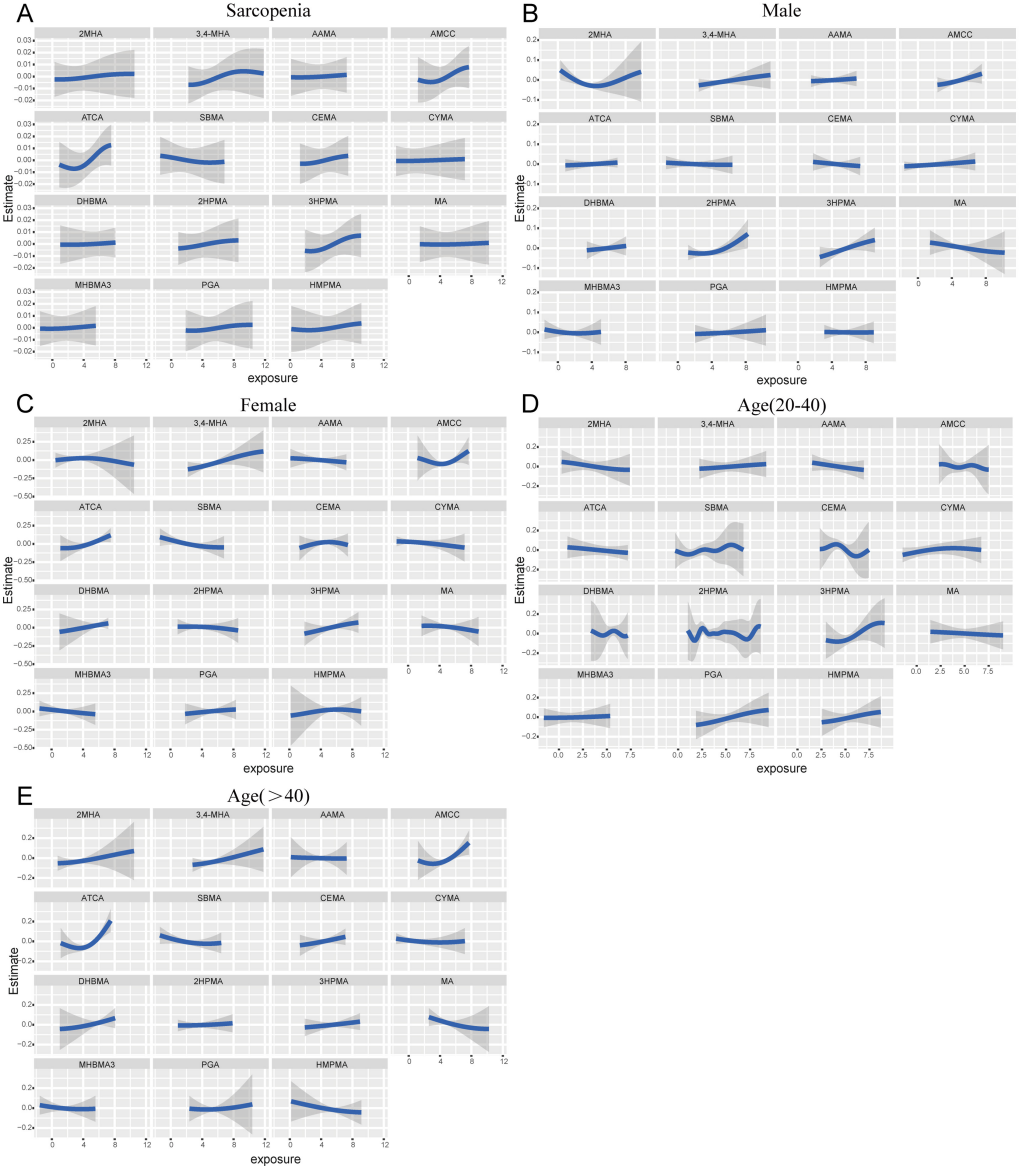
Univariate exposure–response function between each mVOC and the risk of sarcopenia estimated by the BKMR model when the other mVOCs were fixed at 50th percentiles in total participants **(A)**, males **(B)**, females **(C)**, 20 < age ≤ 40 years **(D)**, and age >40 years **(E)**. The model was adjusted for gender, age, race, education, family PIR, marital status, body mass index, smoking status, alcohol drinking, hypertension, diabetes, stroke, white blood cell, ALP, platelet, serum vitamin D, triglycerides, and high-density lipoprotein cholesterol.

**Figure 4:**
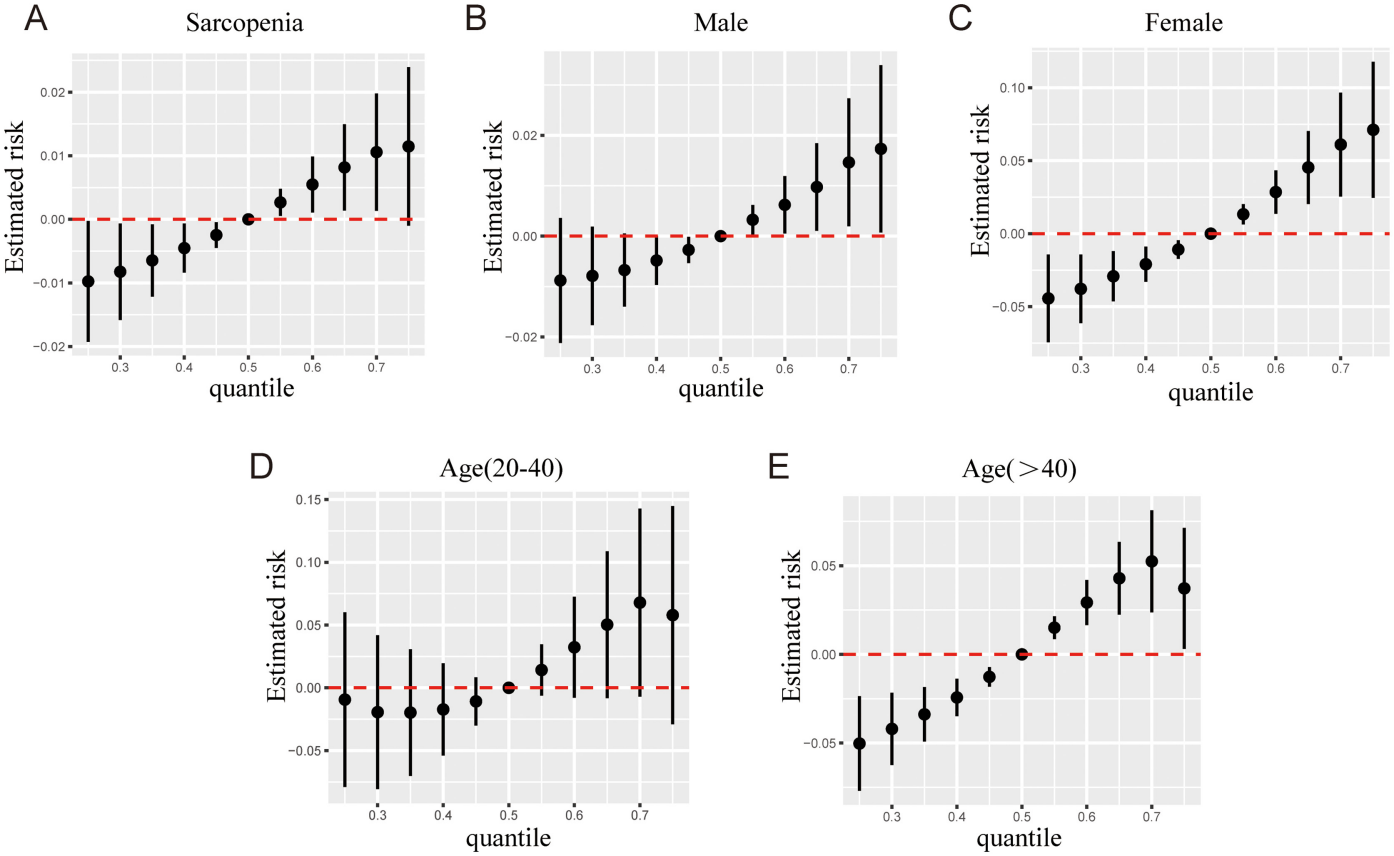
The combined effects of mVOCs mixtures on sarcopenia risk were estimated by BKMR models in total population **(A)**, males **(B)**, females **(C)**, 20 < age ≤ 40 years **(D)**, and age >40 years **(E)**, when the other mVOCs were fixed at the median. The model was adjusted for gender, age, race, education, family PIR, marital status, body mass index, smoking status, alcohol drinking, hypertension, diabetes, stroke, white blood cell, ALP, platelet, serum vitamin D, triglycerides, and high-density lipoprotein cholesterol.

In BKMR analysis, when all other mVOCs were fixed at their median levels, the exposure–response functions revealed 3,4-MHA, AMCC, ATCA, CEMA, 2HPMA, 3HPMA, PGA, and HMPMA were positively associated with sarcopenia (Figure 3). The combined exposure to mVOC mixtures above the 50th percentile is associated with an increased risk of sarcopenia, compared to other mVOCs at the 50th percentile. In the subgroups of males, females, and individuals aged >40, compared to the median, combined exposure to mVOC mixtures above the 50th percentile also exhibited a positive trend with regard to sarcopenia (Figure 4).

Furthermore, CYMA exhibited significant interactions with AMCC, ATCA, CEMA, 3,4-MHA, 3HPMA, and 2HPMA, as indicated in the interaction analysis of 15 mVOCs, suggesting that CYMA may synergistically interact with these compounds to contribute to the development of sarcopenia (Supplementary Figure S3). Subgroup analyses stratified by gender and age revealed that AMCC and 2HPMA were particularly notable in the female subgroup and the 20–40 age subgroup for their interactions with other mVOCs. These interactions, such as between AMCC, 2HPMA, and other mVOCs, were observed more prominently in these specific groups (Supplementary Figures S4–S7). This suggests that the interactivity between mVOC compounds may vary by gender and age, potentially due to biological or hormonal differences that could influence the metabolic processing of these compounds and their synergistic effects on sarcopenia risk.

**Figure 5:**
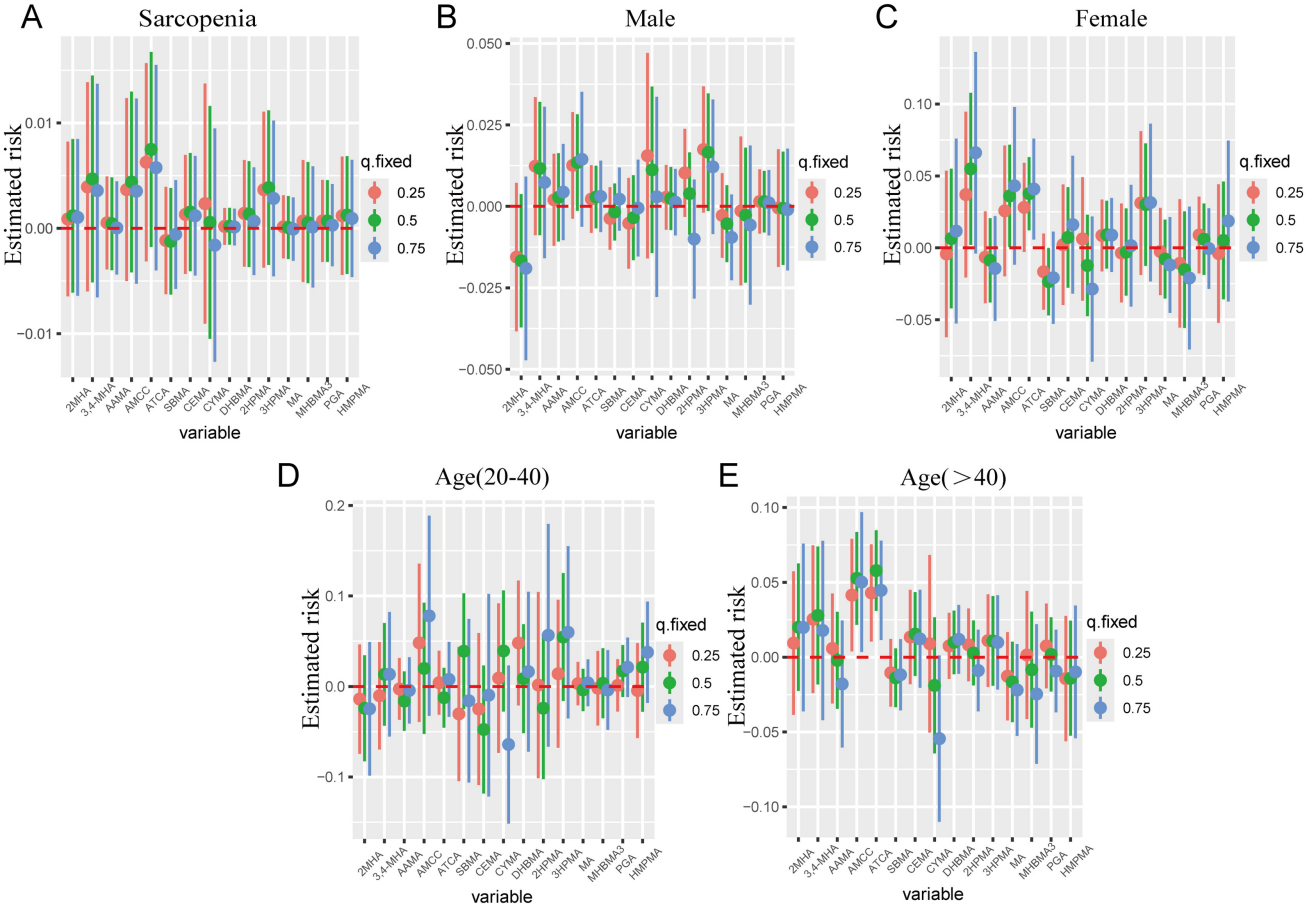
Associations of each mVOC with the risk of sarcopenia estimated by the BKMR model in total participants **(A)**, males **(B)**, females **(C)**, 20 < age ≤ 40 years **(D)**, and age >40 years **(E)**, when other all mVOCs were fixed at their corresponding 25th (red), 50th (green) or 75th (blue) percentile, respectively. All models were adjusted for gender, age, race, education, family PIR, marital status, body mass index, smoking status, alcohol drinking, hypertension, diabetes, stroke, white blood cell, ALP, platelet, serum vitamin D, triglycerides, and high-density lipoprotein cholesterol.

The groupPIP and condPIP values derived from the BKMR regression analysis across different subgroups were presented in Table 3. In total participants, the group with the highest groupPIP was the third group (group PIP = 1.00), in which ATCA had the highest condPIP of 1.00 (Table 3). The group PIP and cond PIP of mVOCs varied between the different subgroups. When the concentrations of other mVOCs were fixed at the 25th, 50th, and 75th percentiles, no metabolites were significantly associated with sarcopenia in the overall population (Figure 5). Furthermore, in the female group, AMCC and ATCA were positively associated with sarcopenia when other mVOCs were fixed at 50th and 75th percentiles, respectively. In the subgroup of individuals aged over 40 years, we observed a positive association between AMCC and ATCA with sarcopenia, while other mVOCs were held constant at the 25th, 50th, and 75th percentiles (Figure 5).

**Table 3:** Posterior inclusion probabilities (PIPs) of mVOCs on sarcopenia in different subgroups within the hierarchical BKMR model.

	**Total participants**			**Male group**			**Female group**		
**mVOCs**	**Group**	**GroupPIP**	**CondPIP**	**Group**	**GroupPIP**	**CondPIP**	**Group**	**GroupPIP**	**CondPIP**
2MHA	1	0.76	0.25	1	0.67	0.16	1	0.03	0.00
3,4-MHA	1	0.76	0.75	1	0.67	0.07	1	0.03	1.00
AAMA	2	0.59	0.07	1	0.67	0.06	2	0.32	0.00
AMCC	2	0.59	0.07	1	0.67	0.00	2	0.32	0.03
ATCA	3	1.00	1.00	2	0.71	0.41	3	0.69	1.00
SBMA	3	1.00	0.00	2	0.71	0.59	3	0.69	0.00
CEMA	2	0.59	0.32	1	0.67	0.16	2	0.32	0.13
CYMA	4	0.85	0.14	3	0.97	0.77	4	0.03	0.00
DHBMA	2	0.59	0.10	1	0.67	0.00	2	0.32	0.25
2HPMA	2	0.59	0.07	4	0.85	1.00	2	0.32	0.00
3HPMA	4	0.85	0.19	3	0.97	0.19	4	0.03	0.00
MA	2	0.59	0.27	1	0.67	0.36	2	0.32	0.25
MHBMA3	4	0.85	0.44	3	0.97	0.04	4	0.03	0.00
PGA	2	0.59	0.10	1	0.67	0.18	2	0.32	0.34
HMPMA	4	0.85	0.24	3	0.97	0.00	4	0.03	1.00
				**Age group 20–40**			**Age group over 40**		
**mVOCs**				**Group**	**GroupPIP**	**CondPIP**	**Group**	**GroupPIP**	**CondPIP**
2MHA				1	0.53	0.87	1	0.01	0.00
3,4-MHA				1	0.53	0.13	1	0.01	1.00
AAMA				2	1.00	0.00	2	0.09	0.00
AMCC				2	1.00	0.00	2	0.09	1.00
ATCA				3	0.19	0.53	3	1.00	1.00
SBMA				3	0.19	0.47	3	1.00	0.00
CEMA				2	1.00	0.00	2	0.09	0.00
CYMA				2	1.00	0.00	2	0.09	0.00
DHBMA				2	1.00	0.00	4	0.01	0.00
2HPMA				4	0.29	1.00	4	0.01	0.00
3HPMA				2	1.00	0.97	2	0.09	0.00
MA				2	1.00	0.00	4	0.01	1.00
MHBMA3				2	1.00	0.00	2	0.09	0.00
PGA				2	1.00	0.00	4	0.01	0.00
HMPMA				2	1.00	0.03	2	0.09	0.00

The mVOCs were grouped based on the correlation matrix.

mVOCs, metabolites of volatile organic compounds; GroupPIP, group posterior inclusion probability; CondPIP, conditional posterior inclusion probability.

### 3.5 Mediation analysis of mediators on the correlation between mVOCs co-exposure and sarcopenia risk

We assessed whether metabolic factors (TG/HDL-C), endocrine factors (vitamin D) and inflammation biomarkers (SII index, WBC, and ALP) mediate the correlation between mVOCs mixtures and sarcopenia. Inflammation biomarkers significantly mediated the positive associations of mVOCs with sarcopenia, with ALP accounting for 8.5% of the mediation, WBC for 5.1%, and SII for 1.9%. However, vitamin D of endocrine factors may exert an inhibitory effect on the relationship between mVOCs and sarcopenia [indirect effect (IE): −0.003, 95% CI: −0.006–0.00, *P* = 0.04], with a mediation proportion of 4.6% (Table 4, Supplementary Figure S8). This finding suggests that higher levels of vitamin D may reduce the detrimental impact of mVOC exposure on sarcopenia risk. This negative mediation effect implies that vitamin D could potentially play a protective role in modulating the adverse effects of mVOC exposure on skeletal muscle health.

**Table 4:** Mediating effect and proportions of metabolic factors, endocrine factors and inflammation biomarkers between mVOCs and the prevalence of sarcopenia.

**Pathways**	**Indirect effect**	**95% CI**	***P*-value**	**Mediation proportions**	**95% CI**	***P*-value**
**Metabolic factors**						
mVOCs → TG/HDL-C → sarcopenia	0.000	−0.001–0.00	0.28	0.2%	−0.022–0.002	0.28
**Endocrine factors**						
mVOCs → Vitamin D → sarcopenia	−0.003	−0.006–0.00	0.04	4.6%	−0.11 to −0.001	0.04
**Inflammation factors**						
mVOCs → SII → sarcopenia	0.001	0.00–0.003	0.01	1.9%	0.004–0.051	0.01
mVOCs → WBC → sarcopenia	0.003	0.001–0.006	0.00	5.1%	0.019–0.11	0.00
mVOCs → ALP → sarcopenia	0.005	0.003–0.009	0.00	8.5%	0.045–0.15	0.00

The mVOCs were grouped based on the correlation matrix.

mVOCs, metabolites of volatile organic compounds; SII, Systemic Immune-Inflammation Index; WBC, White blood cell; ALP, Alkaline phosphatase; GroupPIP, group posterior inclusion probability; CondPIP, conditional posterior inclusion probability.

### 3.6 Analysis of mechanisms and potential targets

**Figure 6:**
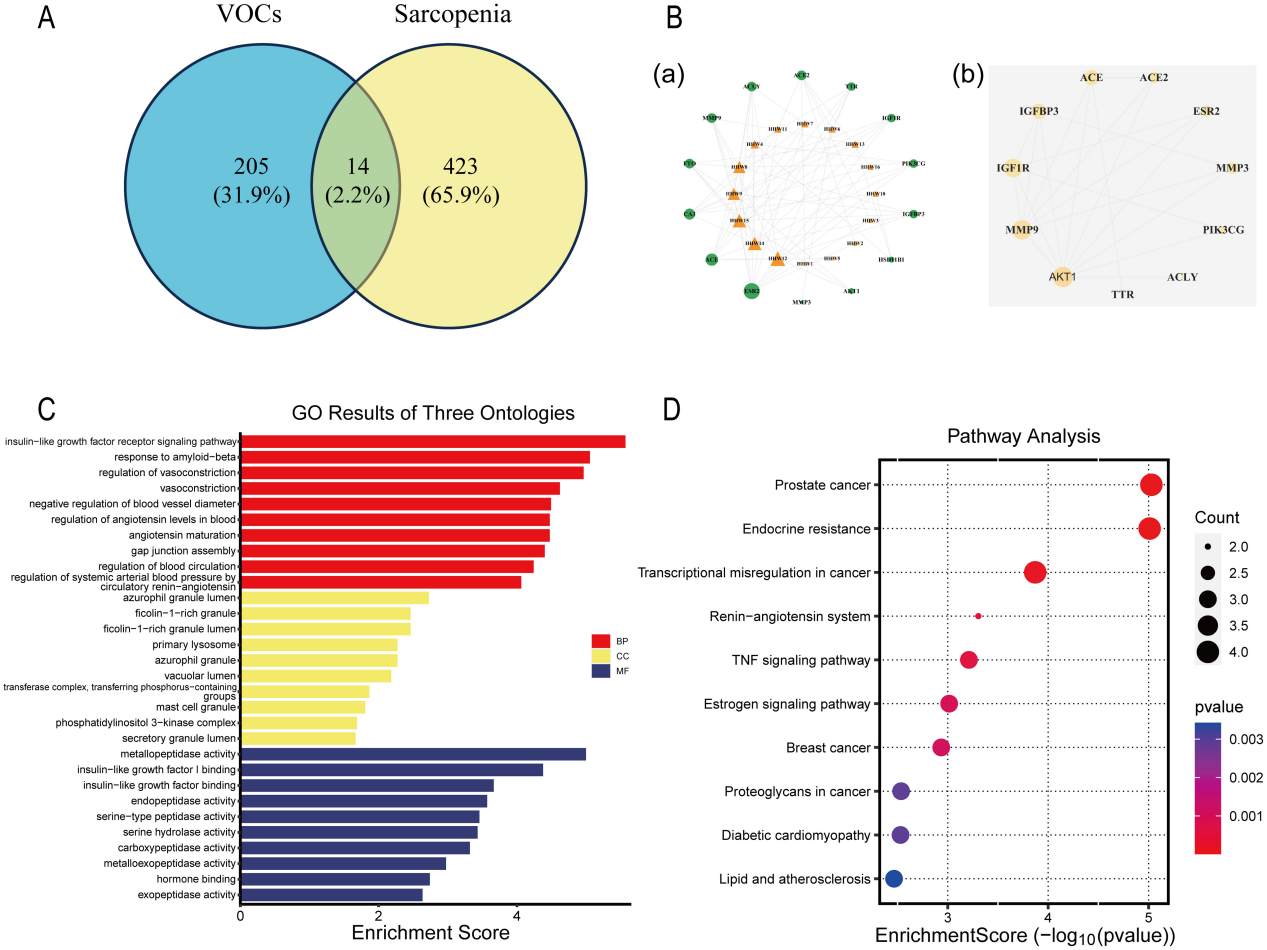
The potential targets and mechanisms by which VOCs contribute to sarcopenia were predicted using a network pharmacology approach. **(A)** A Venn diagram illustrating the number of predicted VOCs targets and the overlapping targets shared with sarcopenia-related targets. **[B**(a)**]** Interaction network between overlapping targets and VOCs. **[B**(b)**]** PPI network of the overlapping targets. **(C)** Bar chart of the GO enrichment analysis. **(D)** Dot plot of the Kyoto Encyclopedia of Genes and Genomes (KEGG) enrichment analysis. VOCs, volatile organic compounds; PPI, Protein-Protein Interaction; GO, Gene Ontology; KEGG, Kyoto Encyclopedia of Genes and Genomes.

We identified a total of 205 target proteins associated with volatile organic compounds (VOCs) from the SwissTargetPrediction database and 423 target proteins associated with sarcopenia from the GeneCards database (Figure 6A). By intersecting the VOCs target proteins with sarcopenia-associated proteins, we identified 14 potential targets involved in VOC-induced sarcopenia [Figure 6B (a)]. The overlapping targets were used to construct a protein-protein interaction (PPI) network using the STRING database, which ultimately led to the identification of 11 core targets associated with VOCs [Figure 6B (b)].

Gene Ontology (GO) analysis was conducted to identify the biological processes (BPs), cellular components (CCs) and molecular functions (MFs) involved in the potential targets (Figure 6C). BPs were predominantly involved in the insulin-like growth factor receptor signaling pathway, response to amyloid-beta, regulation of vasoconstriction, vasoconstriction, negative regulation of blood vessel diameter, regulation of angiotensin levels in blood, angiotensin maturation, gap junction assembly, regulation of blood circulation, and regulation of systemic arterial blood pressure by the circulatory renin-angiotensin system. CCs were Enriched in azurophil granule lumen, ficolin-1-rich granule, ficolin-1-rich granule lumen, primary lysosome, azurophil granule, vacuolar lumen, transferase complex, transferring phosphorus-containing groups, mast cell granule, phosphatidylinositol 3-kinase complex, and secretory granule lumen. MFs were mainly involved in metallopeptidase activity, insulin-like growth factor I binding, insulin-like growth factor binding, endopeptidase activity, serine-type peptidase activity, serine hydrolase activity, carboxypeptidase activity, metalloexopeptidase activity, hormone binding, and exopeptidase activity.

In the KEGG pathway enrichment analysis, the top 10 pathways associated with these targets were identified as: prostate cancer, endocrine resistance, transcriptional misregulation in cancer, renin-angiotensin system, TNF signaling pathway, estrogen signaling pathway, breast cancer, proteoglycans in cancer, diabetic cardiomyopathy, and lipid and atherosclerosis (Figure 6D).

## 4 Discussion

In this study, we employed five different statistical methods to evaluate the individual and combined effects of various mVOCS on sarcopenia. In the multivariable logistic regression analysis, ten mVOCs were found to be significantly correlated with sarcopenia, with ORs ranging from 1.17 to 2.83. Co-exposure to mVOCs showed an increased risk of sarcopenia in the WQS and BKMR model. Those mVOCs included CEMA, AMCC, ATCA, 3,4-MHA and 3HPMA. The RCS analysis revealed a positive linear association between mVOCs and sarcopenia. Moreover, inflammatory factors (SII, WBC, and ALP) partially mediated the positive association between mVOC mixture and sarcopenia, while endocrine factors (vitamin D) inhibited this relationship.

mVOCs have been reported to be associated with several diseases, including diabetes (21), chronic cardiovascular diseases (27), respiratory diseases (43), and cancer (44). To the best of our knowledge, this is the first study to investigate the effects of individual and combined mVOCs on sarcopenia. Our findings contribute to the growing body of literature on environmental pollutants and their potential role in muscle health.

Firstly, regarding the effects of mVOCs on the endocrine system, previous studies have found positive associations between low-level exposure to VOCs, especially HPMMA, and diabetes, insulin resistance (TyG index), fasting glucose (FPG), glycosylated hemoglobin (HbA1c), and insulin levels. Notably, the impact of mVOCs appears to be more pronounced in females and individuals aged 40–59 years (21). This aligns with our findings that mVOCs may disrupt glucose metabolism, potentially contributing to the development of sarcopenia. Additionally, Silan et al. conducted a nested case-control study involving 454 cases of gestational diabetes mellitus (GDM) and 454 matched healthy controls to explore the association between mVOCs and GDM risk. Their results revealed that elevated urinary concentrations of six specific VOCs were significantly associated with an increased risk of GDM, with each quartile increase in exposure correlating with a 19%−27% increase in risk (45). Furthermore, numerous studies have confirmed a strong relationship between the development of diabetes and an elevated risk of sarcopenia (46, 47), which indirectly explains our findings that mVOCs significantly promote the onset of sarcopenia, potentially through the disruption of glucose metabolism regulation. Furthermore, a cross-sectional study of 3,478 participants found that exposure to both individual and combined mVOCs was associated with reduced bone mineral density in U.S. adults (48). Osteoporosis is a well-established contributor to sarcopenia (49), which may represent another potential mechanism through which mVOCs elevate the risk of sarcopenia.

Second, regarding the effects of mVOCs on the immune system, dimethylformamide (DMF), a precursor of AMCC, has been reported to significantly impair lung function, with C-reactive protein (CRP) mediating this process (50). In a prospective study, Schaap et al. (51) found that IL-6 and CRP were associated with the increased risk of muscle strength loss in older adults after adjusting for confounders. Moreover, a cross-sectional study involving individuals aged 90 years and older revealed that interleukin-6 (IL-6), interleukin-1 receptor antagonist (IL-1Ra), and C-reactive protein (CRP) were correlated with the risk of sarcopenia (52). These findings were consistent with our study, indicating that inflammatory mediators play a crucial role in the association between mVOCs and the risk of sarcopenia.

However, the biological mechanisms by which VOCs contribute to sarcopenia remain unclear. Inflammation and endocrine dysregulation may be significant factors in the development of the sarcopenia (53, 54).

VOCs enter the body through the oral cavity, gastrointestinal tract, or respiratory system. Prolonged exposure to these compounds results in the generation of reactive oxygen species (ROS) in human alveolar epithelial cells. Interestingly, there is no corresponding increase in free radical scavengers, such as antioxidants. The accumulation of ROS subsequently leads to the activation of nuclear factor kappa B (NF-κB), which stimulates the expression of specific genes involved in the synthesis of inflammatory proteins. This process facilitates the recruitment of various immune cells, including leukocytes and macrophages, to sites of oxidative stress. The subsequent cascade amplifies the release of pro-inflammatory cytokines, such as tumor necrosis factor alpha (TNF-α), interleukin-6 (IL-6), and interferon-gamma. These dysregulated inflammatory mediators contribute to metabolic disorders, such as insulin resistance, and can infiltrate muscle tissue, promoting muscle loss and ultimately leading to the development of sarcopenia.

VOCs can alter hormone levels by mimicking or disrupting the functions of endogenous hormones, particularly estrogen (55). This hormonal imbalance may directly affect skeletal metabolism by disrupting the balance between osteoblasts (responsible for bone formation) and osteoclasts (responsible for bone resorption), ultimately leading to osteoporosis. Additionally, VOCs can exert direct toxicity on osteoblasts, inhibiting their function and reducing the synthesis of bone matrix (56). The diminished function of osteoblasts adversely impacts bone formation and maintenance, resulting in decreased bone density and the eventual development of osteoporosis. Bone tissue regulates muscle metabolism by secreting bioactive factors. Osteocalcin, secreted by osteoblasts, plays a crucial role in muscle metabolic regulation. It activates the G protein-coupled receptor signaling pathway, promoting Akt phosphorylation, which leads to the translocation of glucose transporter GLUT4 to the plasma membrane, thereby enhancing glucose uptake in muscle cells (57). Moreover, osteocalcin signaling increases the phosphorylation of AMPK and mTOR, as well as the activity of CPT1B in muscle fibers, facilitating the catabolism of fatty acids and the synthesis of proteins (58), ultimately improving muscular energy metabolism. In conditions of osteoporosis, reduced osteoblast activity leads to a decline in these processes, resulting in decreased muscle synthesis and the onset of sarcopenia. In the present study, we found that SII, WBC, and ALP were involved in the positive correlation between mVOCs and the incidence of sarcopenia, with mediating contributions of 1.9%, 5.1%, and 8.5%, respectively. Therefore, we hypothesize that exposure to VOCs increases the incidence of sarcopenia by promoting inflammation. Interestingly, in contrast to these pro-inflammatory mediators, the endocrine factor vitamin D showed a negative mediating effect in the relationship between mVOCs and sarcopenia, with a mediation ratio of 4.6%. This negative effect suggests that vitamin D may mitigate the harmful impact of mVOCs on sarcopenia risk. The result is consistent with the well-established role of vitamin D in bone and muscle health. Vitamin D is known to regulate calcium and phosphate metabolism, and deficiency in vitamin D has been linked to muscle weakness and sarcopenia. In this context, our findings indicate that higher levels of vitamin D could potentially attenuate the pro-inflammatory effects of VOCs, providing a protective mechanism against sarcopenia. Moreover, the GO and KEGG enrichment analyses revealed significant overlap in the targets between VOCs and sarcopenia in endocrine pathways, further supporting the notion that vitamin D might act as a key modulator in this context. Taken together, these results underscore the complex interplay between environmental pollutants, inflammatory processes, and endocrine factors, highlighting vitamin D's potential as a protective factor against sarcopenia in individuals exposed to mVOCs.

In this study, subgroup analyses stratified by age and gender revealed that the effects of individual mVOC on sarcopenia were not entirely consistent across different subgroups. However, there were mVOCs associated with the outcome in each subgroup. There was an observed association between the co-exposure to mVOCs and sarcopenia, particularly pronounced in males, females, and those over 40 years of age, whereas this significance was not evident in younger groups. Age seems to modulate the negative correlation between mVOCs and sarcopenia. The underlying reasons for this age-related disparity remain unclear. It may be due to the decline in metabolic rate, muscle mass and strength that often accompanies aging, leading to an increased accumulation of mVOCs in the body, which subsequently reduces testosterone levels (59). Satellite cells, the stem cells of skeletal muscle, play a crucial role in muscle growth and repair. Testosterone enhances the survival of these satellite cells by increasing the expression of androgen receptors on their surface, thereby activating the phosphoinositide 3-kinase/Akt signaling pathway, which inhibits the activity of pro-apoptotic factors (60–63). Additionally, testosterone increases the protein expression and activity of mitochondrial manganese superoxide dismutase, which protects the mitochondrial membrane potential and inhibits the opening of the mitochondrial permeability transition pore induced by H_2_O_2_. In contrast, the increase in VOC metabolites, by reducing endogenous testosterone levels, disrupts satellite cell activity and consequently promotes the development of sarcopenia.

This study possesses several strengths. First, participants included in this study were derived from the nationally representative NHANES survey with a large sample size. Second, this is the first study to investigate the individual and joint effect of several mVOCs on the risk of sarcopenia through multiple statistical models, including the WQS model, and BKMR model. These models address the issue of collinearity among mVOC components and robustly identify the compounds most strongly associated with sarcopenia risk. In addition, the study explores the relationship between mVOC mixtures and sarcopenia across different subgroups, and examines the mediating effects of metabolic, endocrine, and inflammatory factors on the VOC-sarcopenia association.

However, there remained some limitations to our study. First, this study utilized a cross-sectional design, limiting our ability to infer a causal relationship between mVOCs and sarcopenia. Therefore, further cohort studies are required to validate the association between combined mVOC exposure and sarcopenia, as well as the roles of inflammatory and metabolic factors in this relationship. Additionally, despite the comprehensive adjustments made for potential confounders, several unmeasured factors could still influence the observed associations. For example, occupational exposures, physical activity, and dietary habits are known to impact both VOCs exposure and sarcopenia but were not directly accounted for in our analysis. Although we included a range of covariates, these unmeasured confounders may have introduced residual bias, which could influence the strength and direction of the associations observed in this study. Third, the exact mechanisms by which mVOCs contribute to the development of sarcopenia remain unclear, and further animal studies are needed to explore the underlying biological mechanisms. The network pharmacology analysis identified potential targets and pathways, but these findings require experimental validation. While we have used databases such as DrugBank, SwissTargetPrediction, and STRING to identify potential pharmacological targets, and conducted pathway enrichment analysis, further *in vitro* and *in vivo* experiments are necessary to confirm the biological relevance of these targets and pathways in the context of sarcopenia. Future experiments will focus on validating the core targets identified through network pharmacology, such as those involved in endocrine signaling and immune response, to better understand how VOC exposure may affect sarcopenia development.

In addition, while the sample size in this study is relatively small (145 sarcopenia patients), which could potentially limit statistical power, we employed rigorous variable selection and utilized multiple statistical methods to validate the robustness of our findings, including WQS and BKMR regression models. Our results consistently showed an association between VOC exposure and sarcopenia, strengthening the reliability of our conclusions across different analytic techniques. However, we acknowledge that future studies with larger longitudinal samples are warranted to further confirm these preliminary findings. Finally, the study participants were drawn from an adult population in the United States, and the generalizability of the findings to other populations and regions requires further investigation.

## 5 Conclusion

In summary, our study provided preliminary evidence that exposure to individual and mixed VOCs were positively associated with the risk of sarcopenia, particularly among older adults. Alkaline phosphatase (ALP), white blood cell count (WBC), the systemic immune-inflammation index (SII), and vitamin D were identified as mediators in the relationship between mixed mVOCs and sarcopenia. Endocrine resistance pathway was the underlying mechanism.

## Data Availability

The original contributions presented in the study are included in the article/Supplementary material, further inquiries can be directed to the corresponding authors.

## Supplementary material

Supplementary Figure 1 Spearman correlation coefficient among the fifteen ln-transformed volatile organic compound metabolites.

Supplementary Figure 2 Restricted cubic spline plots of the association between ln-transformed mVOCs and sarcopenia. The model was adjusted for gender, age, race, education, family PIR, marital status, body mass index, smoking status, alcohol drinking, hypertension, diabetes, stroke, white blood cell, ALP, platelet, serum vitamin D, triglycerides, and high-density lipoprotein cholesterol.

Supplementary Figure 3 The interaction of each mVOC in total participants. The model was adjusted for gender, age, race, education, family PIR, marital status, body mass index, smoking status, alcohol drinking, hypertension, diabetes, stroke, white blood cell, ALP, platelet, serum vitamin D, triglycerides, and high-density lipoprotein cholesterol.

Supplementary Figure 4 Interactions among each mVOC within the male subgroup. The model was adjusted for gender, age, race, education, family PIR, marital status, body mass index, smoking status, alcohol drinking, hypertension, diabetes, stroke, white blood cell, ALP, platelet, serum vitamin D, triglycerides, and high-density lipoprotein cholesterol.

Supplementary Figure 5 Interactions among each mVOC within the female subgroup. The model was adjusted for gender, age, race, education, family PIR, marital status, body mass index, smoking status, alcohol drinking, hypertension, diabetes, stroke, white blood cell, ALP, platelet, serum vitamin D, triglycerides, and high-density lipoprotein cholesterol.

Supplementary Figure 6 Interactions among each mVOC within the subgroup aged 20 to 40 years. The model was adjusted for gender, age, race, education, family PIR, marital status, body mass index, smoking status, alcohol drinking, hypertension, diabetes, stroke, white blood cell, ALP, platelet, serum vitamin D, triglycerides, and high-density lipoprotein cholesterol.

Supplementary Figure 7 Interactions among each mVOC within the subgroup aged over 40 years. The model was adjusted for gender, age, race, education, family PIR, marital status, body mass index, smoking status, alcohol drinking, hypertension, diabetes, stroke, white blood cell, ALP, platelet, serum vitamin D, triglycerides, and high-density lipoprotein cholesterol.

Supplementary Figure 8 The mediating roles of metabolic factors, endocrine factors and inflammation biomarkers in the association between VOCs co-exposure and sarcopenia. **(A)** TG/HDL-C, triglycerides/high-density lipoprotein cholesterol; **(B)** Vitamin D; **(C)** SII, the systemic immune-inflammation index; **(D)** WBC, white blood cell count; **(E)** ALP, alkaline phosphatase. All models were adjusted for gender, age, race, education, family PIR, marital status, body mass index, smoking status, alcohol drinking, hypertension, diabetes, stroke, white blood cell, ALP, platelet, serum vitamin D, triglycerides, and high-density lipoprotein cholesterol. mVOCs, metabolites of volatile organic compounds (VOCs). IE, indirect effect; DE, direct effect; Proportion of mediation = IE / (DE + IE). ^*^*P* < 0.05.

Supplementary Table 1 Association of continuous urine 2MHA with sarcopenia risk in all participants after adjusting for all covariates.

Supplementary Table 2 Association of categorical urine 2MHA with sarcopenia risk in all participants after adjusting for all covariates.

Supplementary Table 3 Association of continuous urine 3,4-MHA with sarcopenia risk in all participants after adjusting for all covariates.

Supplementary Table 4 Association of categorical urine 3,4-MHA with sarcopenia risk in all participants after adjusting for all covariates.

Supplementary Table 5 Association of continuous urine AAMA with sarcopenia risk in all participants after adjusting for all covariates.

Supplementary Table 6 Association of categorical urine AAMA with sarcopenia risk in all participants after adjusting for all covariates.

Supplementary Table 7 Association of continuous urine AMCC with sarcopenia risk in all participants after adjusting for all covariates.

Supplementary Table 8 Association of categorical urine AMCC with sarcopenia risk in all participants after adjusting for all covariates.

Supplementary Table 9 Association of continuous urine ATCA with sarcopenia risk in all participants after adjusting for all covariates.

Supplementary Table 10 Association of categorical urine ATCA with sarcopenia risk in all participants after adjusting for all covariates.

Supplementary Table 11 Association of continuous urine SBMA with sarcopenia risk in all participants after adjusting for all covariates.

Supplementary Table 12 Association of categorical urine SBMA with sarcopenia risk in all participants after adjusting for all covariates.

Supplementary Table 13 Association of continuous urine CEMA with sarcopenia risk in all participants after adjusting for all covariates.

Supplementary Table 14 Association of categorical urine CEMA with sarcopenia risk in all participants after adjusting for all covariates.

Supplementary Table 15 Association of continuous urine CYMA with sarcopenia risk in all participants after adjusting for all covariates.

Supplementary Table 16 Association of categorical urine CYMA with sarcopenia risk in all participants after adjusting for all covariates.

Supplementary Table 17 Association of continuous urine DHBMA with sarcopenia risk in all participants after adjusting for all covariates.

Supplementary Table 18 Association of categorical urine DHBMA with sarcopenia risk in all participants after adjusting for all covariates.

Supplementary Table 19 Association of continuous urine 2HPMA with sarcopenia risk in all participants after adjusting for all covariates.

Supplementary Table 20 Association of categorical urine 2HPMA with sarcopenia risk in all participants after adjusting for all covariates.

Supplementary Table 21 Association of continuous urine 3HPMA with sarcopenia risk in all participants after adjusting for all covariates.

Supplementary Table 22 Association of categorical urine 3HPMA with sarcopenia risk in all participants after adjusting for all covariates.

Supplementary Table 23 Association of continuous urine MA with sarcopenia risk in all participants after adjusting for all covariates.

Supplementary Table 24 Association of categorical urine MA with sarcopenia risk in all participants after adjusting for all covariates.

Supplementary Table 25 Association of continuous urine MHBMA3 with sarcopenia risk in all participants after adjusting for all covariates.

Supplementary Table 26 Association of categorical urine MHBMA3 with sarcopenia risk in all participants after adjusting for all covariates.

Supplementary Table 27 Association of continuous urine PGA with sarcopenia risk in all participants after adjusting for all covariates.

Supplementary Table 28 Association of categorical urine PGA with sarcopenia risk in all participants after adjusting for all covariates.

Supplementary Table 29 Association of continuous urine HMPMA with sarcopenia risk in all participants after adjusting for all covariates.

Supplementary Table 30 Association of categorical urine HMPMA with sarcopenia risk in all participants after adjusting for all covariates.
