# Correction: Volatile organic compounds exposure associated with sarcopenia in US adults from NHANES 2011–2018

**DOI:** 10.3389/fpubh.2025.1770173

**Published:** 2026-01-12

**Authors:** Pangbo Wang, Wei Chen, Hongwei Fang, Liwei Xu, Jun Zhao, Jing Huang

**Affiliations:** 1 Hand and Foot Microsurgery, NO. 946 Hospital of PLA Land Force, Yining, Xinjiang, China; 2 Department of Laboratory Medicine, NO. 946 Hospital of PLA Land Force, Yining, Xinjiang, China; 3 Trauma Neurosurgery, NO. 946 Hospital of PLA Land Force, Yining, Xinjiang, China; 4 Department of Neurosurgery and State Key Laboratory of Trauma, Burn and Combined Injury, Southwest Hospital, Chongqing, China; 5 Chongqing Key Laboratory of Precision Neuromedicine and Neuroregenaration, Third Military Medical University (Army Medical University), Chongqing, China; 6 School of Nursing, Peking University, Beijing, China

**Keywords:** volatile organic compounds, sarcopenia, weighted quantile sum regression, Bayesian Kernel machine regression, network pharmacology analysis

Affiliations were erroneously assigned in the original version of the article. The correct author affiliations are now provided as follows:

**Pangbo Wang** ^1,4,5*^ **, Wei Chen** ^**2**^ **, Hongwei Fang** ^**3**^ **, Liwei Xu** ^**1**^ **, Jun Zhao** ^3*^ **, Jing Huang** ^6*^

^1^Hand and Foot Microsurgery, NO. 946 Hospital of PLA Land Force, Yining, Xinjiang, China

^2^Department of Laboratory Medicine, NO. 946 Hospital of PLA Land Force, Yining, Xinjiang, China

^3^Trauma Neurosurgery, NO. 946 Hospital of PLA Land Force, Yining, Xinjiang, China

^4^Department of Neurosurgery and State Key Laboratory of Trauma, Burn and Combined Injury, Southwest Hospital, Chongqing, China

^5^Chongqing Key Laboratory of Precision Neuromedicine and Neuroregenaration, Third Military Medical University (Army Medical University), Chongqing, China

^6^School of Nursing, Peking University, Beijing, China

The original version of this article has been updated.

