# Supplementary figures and images for: Volatile organic compounds exposure associated with sarcopenia in US adults from NHANES 2011–2018
Source: Front Public Health. 2025 Jul 15;13:1613435. doi: 10.3389/fpubh.2025.1613435 (PMC12303945; doi:10.3389/fpubh.2025.1613435)

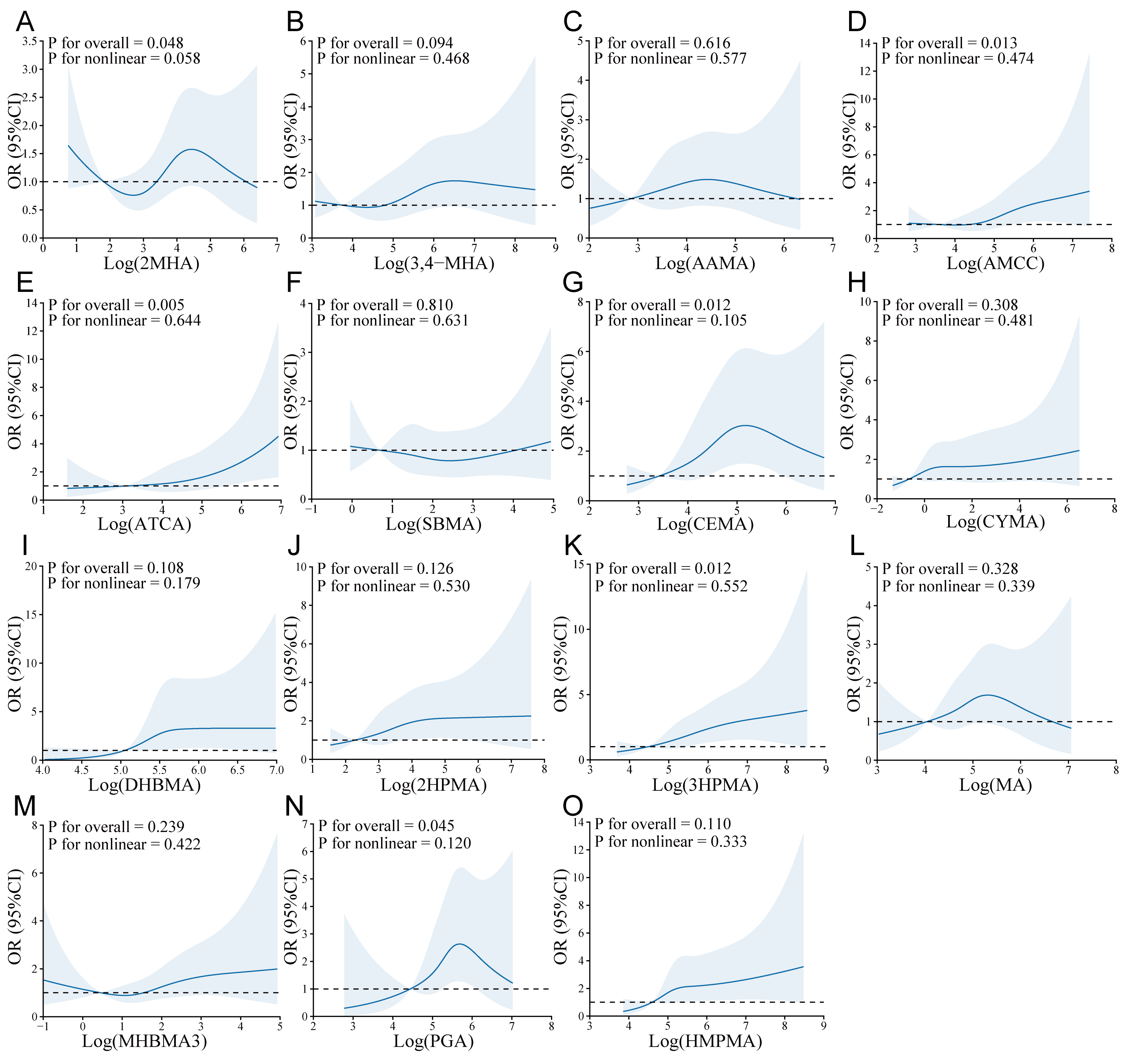

Supplement: Supplementary Figure 1 — Spearman correlation coefficient among the fifteen ln-transformed volatile organic compound metabolites. [file Image_1.tif]

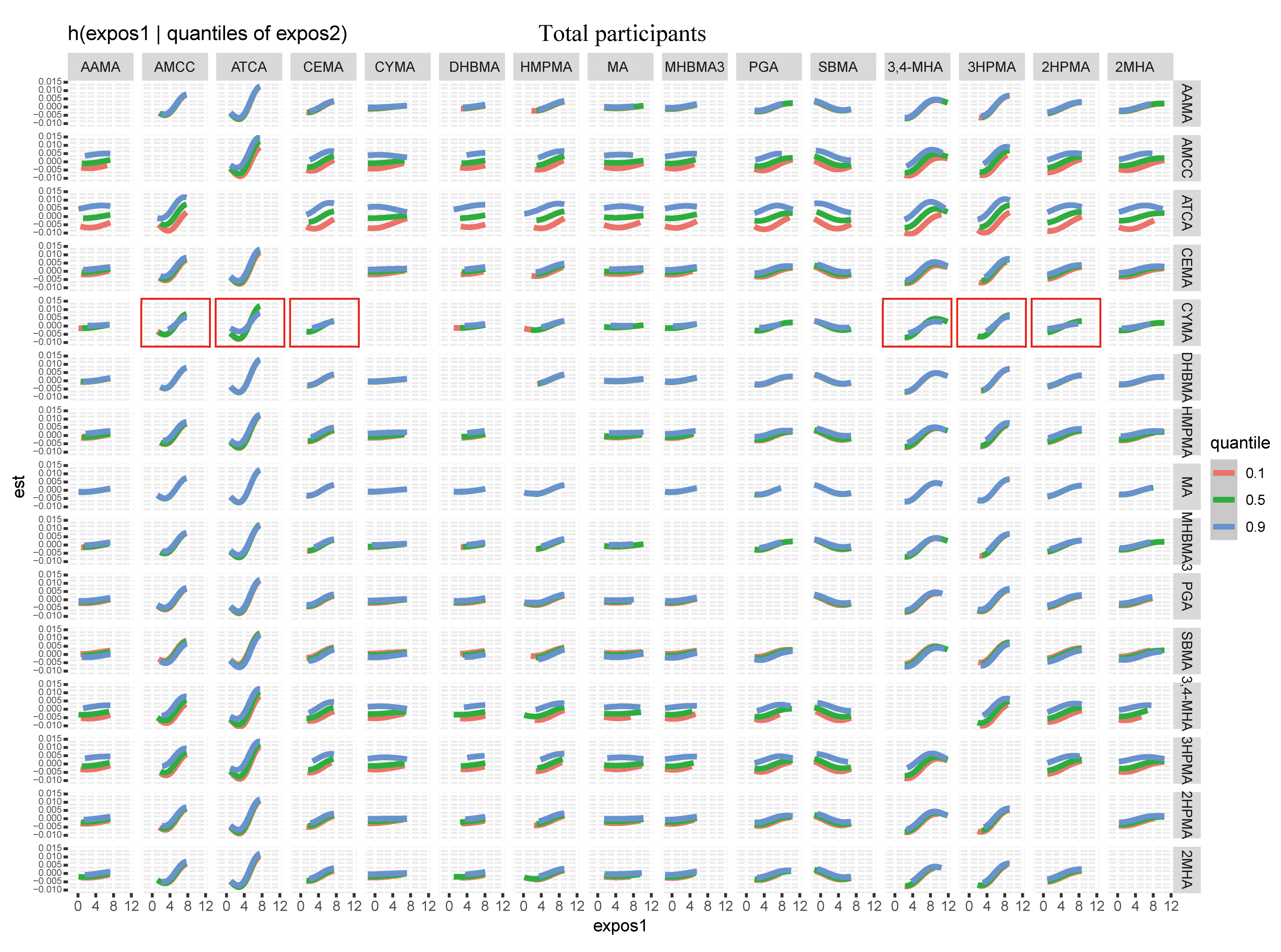

Supplement: Supplementary Figure 2 — Restricted cubic spline plots of the association between ln-transformed mVOCs and sarcopenia. The model was adjusted for gender, age, race, education, family PIR, marital status, body mass index, smoking status, alcohol drinking, hypertension, diabetes, stroke, white blood cell, ALP, platelet, serum vitamin D, triglycerides, and high-density lipoprotein cholesterol. [file Image_2.tif]

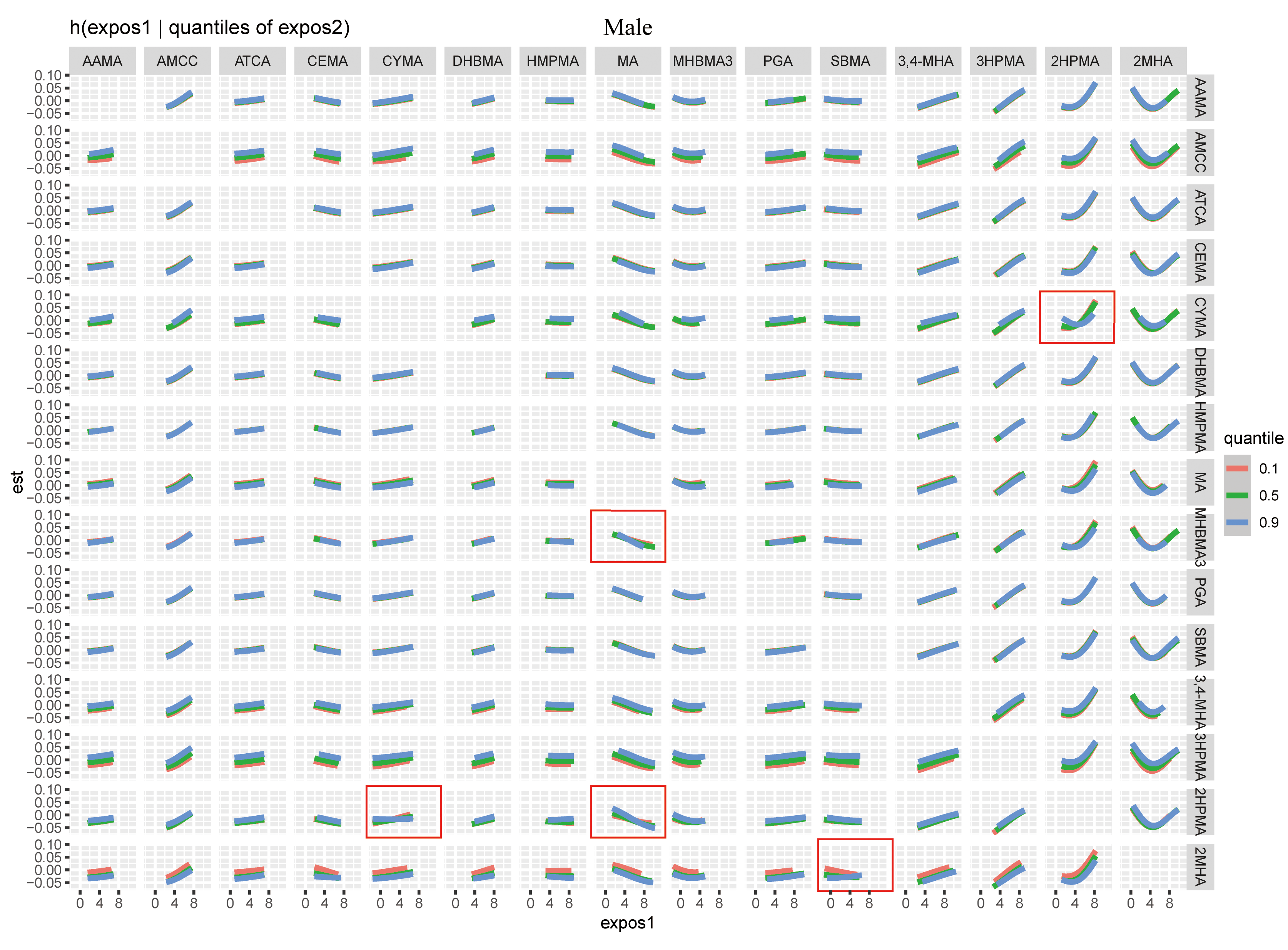

Supplement: Supplementary Figure 3 — The interaction of each mVOC in total participants. The model was adjusted for gender, age, race, education, family PIR, marital status, body mass index, smoking status, alcohol drinking, hypertension, diabetes, stroke, white blood cell, ALP, platelet, serum vitamin D, triglycerides, and high-density lipoprotein cholesterol. [file Image_3.tif]

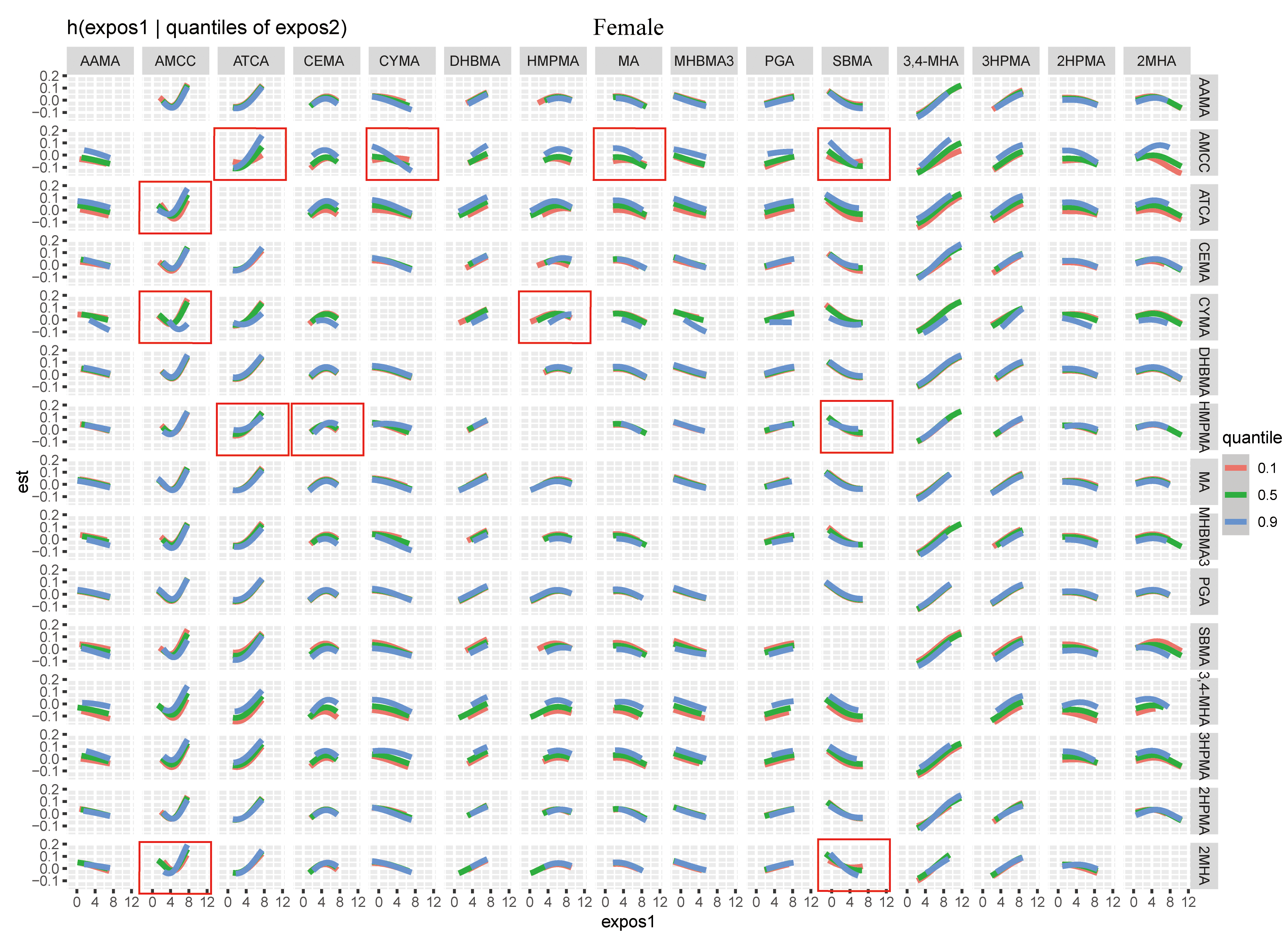

Supplement: Supplementary Figure 4 — Interactions among each mVOC within the male subgroup. The model was adjusted for gender, age, race, education, family PIR, marital status, body mass index, smoking status, alcohol drinking, hypertension, diabetes, stroke, white blood cell, ALP, platelet, serum vitamin D, triglycerides, and high-density lipoprotein cholesterol. [file Image_4.tif]

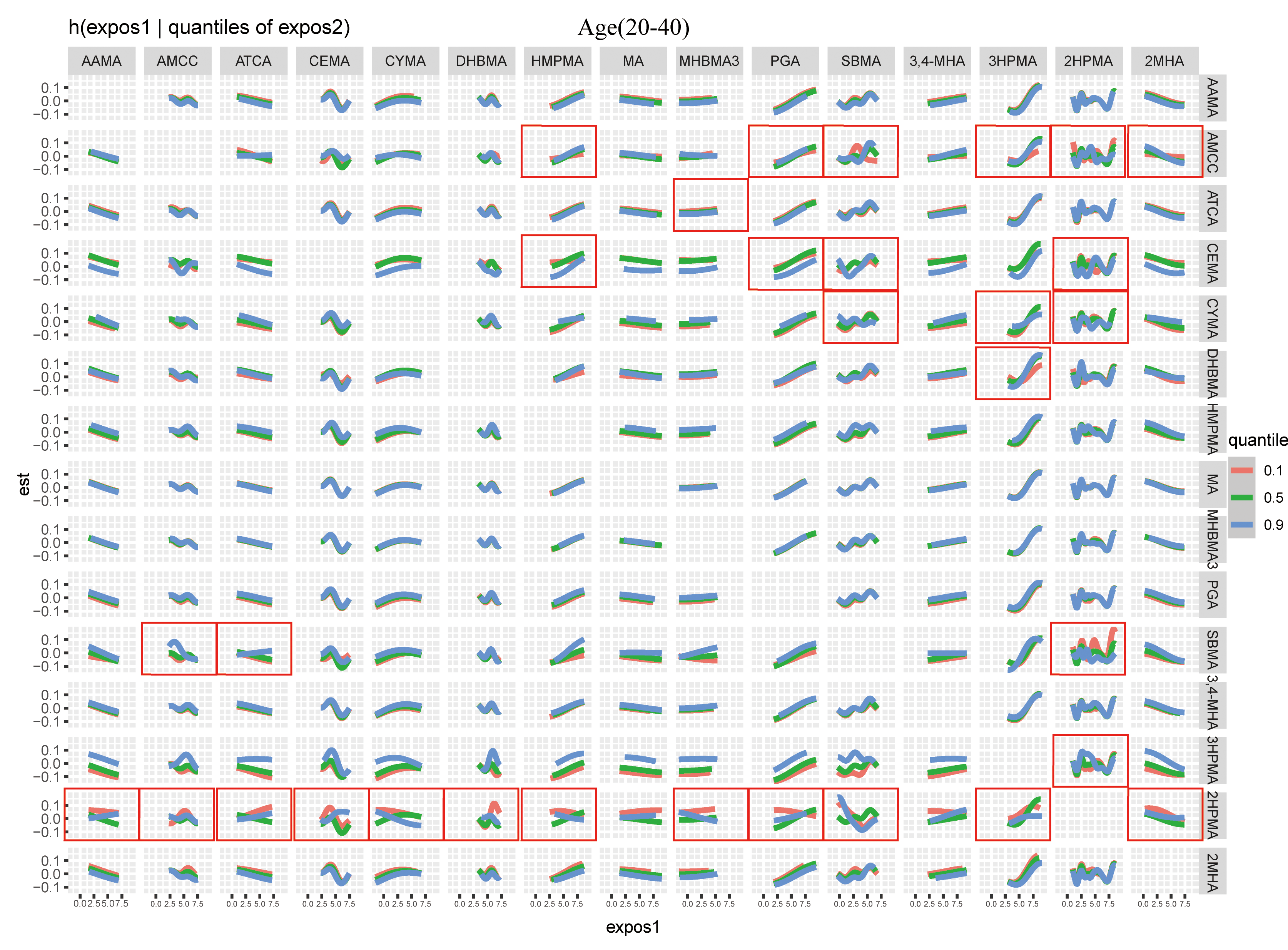

Supplement: Supplementary Figure 5 — Interactions among each mVOC within the female subgroup. The model was adjusted for gender, age, race, education, family PIR, marital status, body mass index, smoking status, alcohol drinking, hypertension, diabetes, stroke, white blood cell, ALP, platelet, serum vitamin D, triglycerides, and high-density lipoprotein cholesterol. [file Image_5.tif]

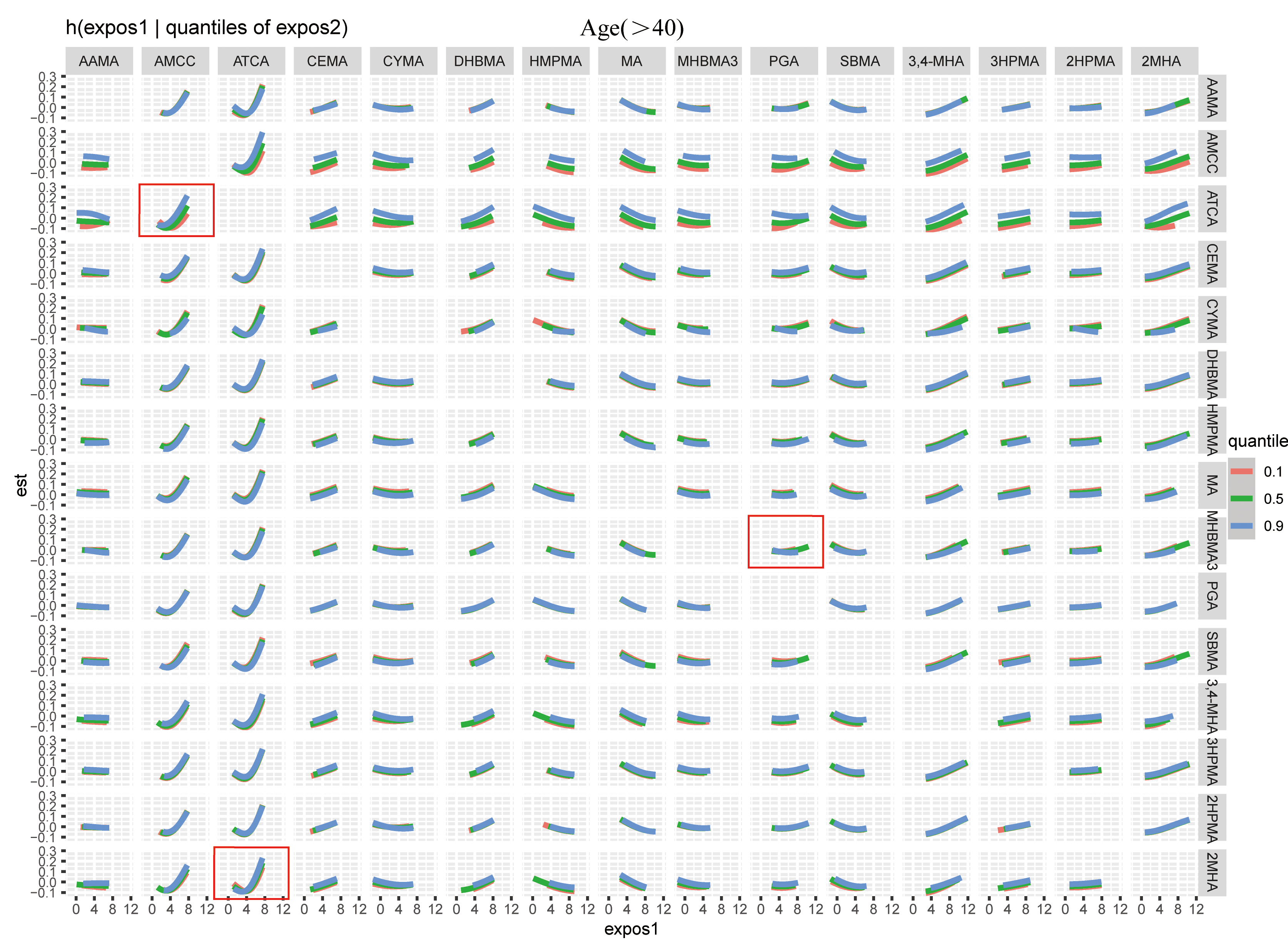

Supplement: Supplementary Figure 6 — Interactions among each mVOC within the subgroup aged 20 to 40 years. The model was adjusted for gender, age, race, education, family PIR, marital status, body mass index, smoking status, alcohol drinking, hypertension, diabetes, stroke, white blood cell, ALP, platelet, serum vitamin D, triglycerides, and high-density lipoprotein cholesterol. [file Image_6.tif]

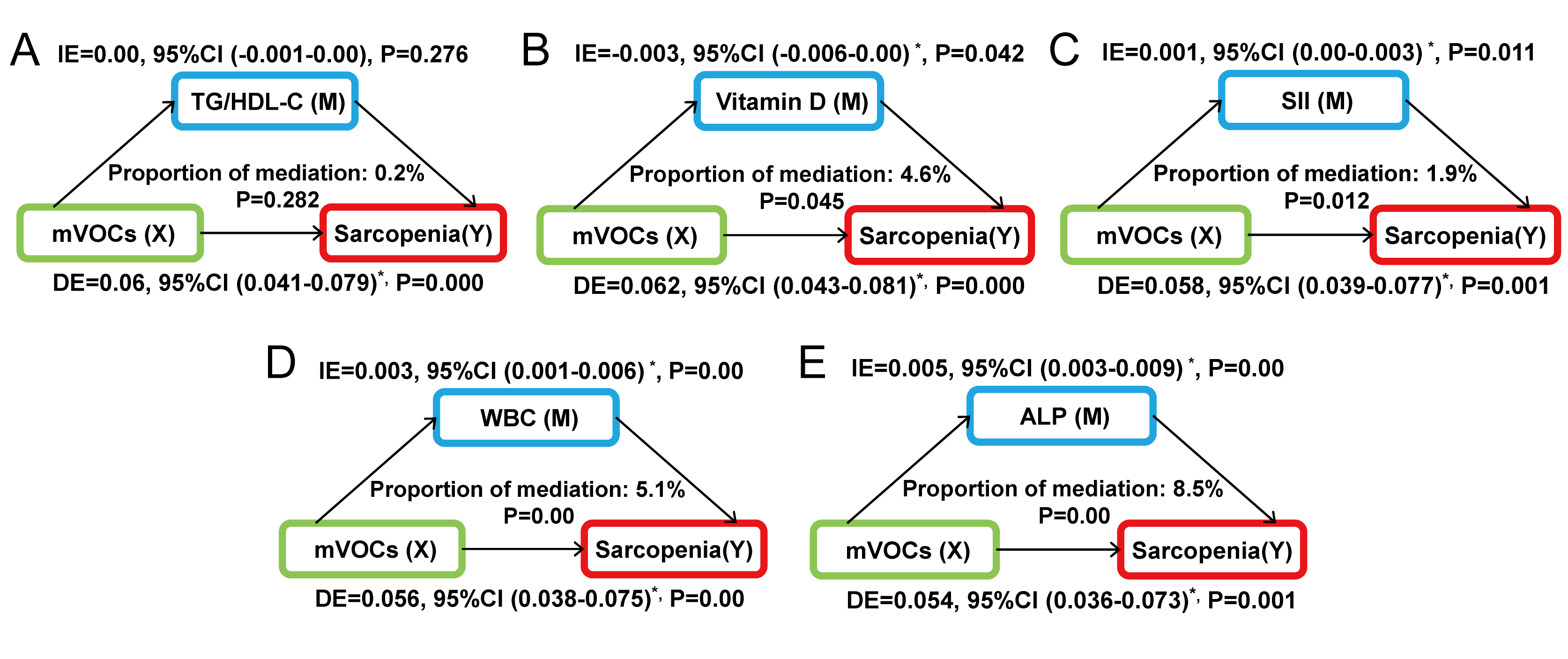

Supplement: Supplementary Figure 7 — Interactions among each mVOC within the subgroup aged over 40 years. The model was adjusted for gender, age, race, education, family PIR, marital status, body mass index, smoking status, alcohol drinking, hypertension, diabetes, stroke, white blood cell, ALP, platelet, serum vitamin D, triglycerides, and high-density lipoprotein cholesterol. [file Image_7.tif]

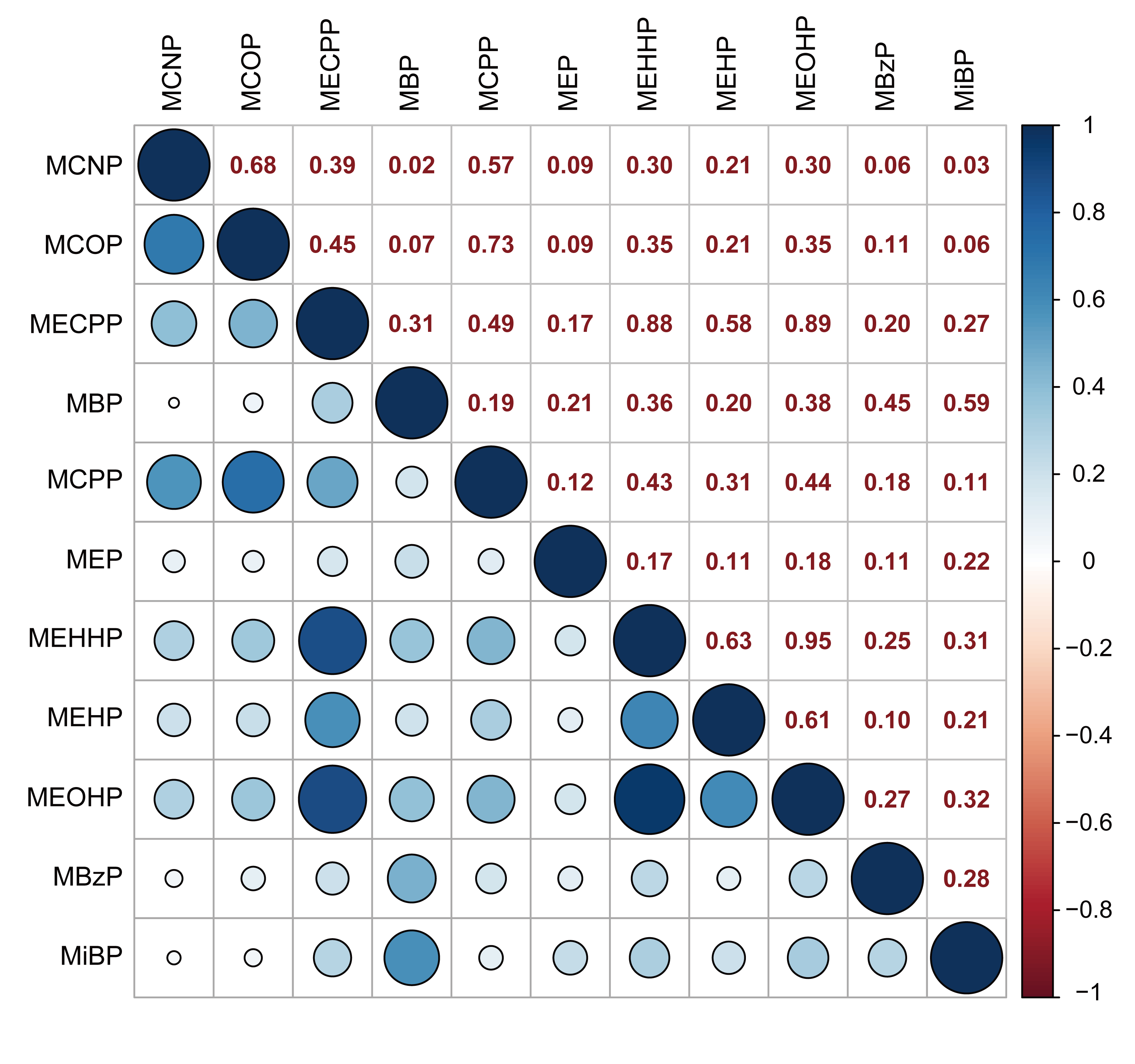

Supplement: Supplementary Figure 8 — The mediating roles of metabolic factors, endocrine factors and inflammation biomarkers in the association between VOCs co-exposure and sarcopenia. (A) TG/HDL-C, triglycerides/high-density lipoprotein cholesterol; (B) Vitamin D; (C) SII, the systemic immune-inflammation index; (D) WBC, white blood cell count; (E) ALP, alkaline phosphatase. All models were adjusted for gender, age, race, education, family PIR, marital status, body mass index, smoking status, alcohol drinking, hypertension, diabetes, stroke, white blood cell, ALP, platelet, serum vitamin D, triglycerides, and high-density lipoprotein cholesterol. mVOCs, metabolites of volatile organic compounds (VOCs). IE, indirect effect; DE, direct effect; Proportion of mediation = IE / (DE + IE). *P < 0.05. [file Image_8.tif]
