# Supplementary material for: Volatile organic compounds exposure associated with sarcopenia in US adults from NHANES 2011–2018
Source: Front Public Health. 2025 Jul 15;13:1613435. doi: 10.3389/fpubh.2025.1613435 (PMC12303945; doi:10.3389/fpubh.2025.1613435)
Supplement: Supplementary Table 1 — Association of continuous urine 2MHA with sarcopenia risk in all participants after adjusting for all covariates. [file Data_Sheet_1.zip › Supplementary R scripts.docx]

1. **Weighted quantile sum (WQS) regression R Code:**

# Clear all results

rm(list = ls())

# Set working directory

setwd("C:/Users/lenovo/Desktop") # Set the data directory

# Install and load necessary package

install.packages("gWQS")

library(gWQS)

library(data.table)

# Read the dataset

a <- fread("data.csv") # It is important to load all environmental exposure data together

# View dataset columns

names(a)

# Define the columns corresponding to the toxic chemicals

toxic_chems <- names(a)[20:34]

# Perform the weighted quantile sum regression

results <- gwqs(RA ~ wqs + age + race + gender + education_level +

marital_status + BMI + smoking_status + alcohol_drinking + hypertension +

stroke + diabetes + WBC + ALP + platelet + vitamin_D + triglycerides + HDL_C,

mix_name = toxic_chems, # Specify the mixture dataset

data = a, q = 4, validation = 0.6, b = 100, # Original dataset and number of bootstrap samples

b1_pos = TRUE, b_constr = FALSE, family = "binomial", # Define direction and type of outcome variable

seed = 2000, plots = TRUE, tables = TRUE) # Set seed for reproducibility and enable plots/tables

# Summarize the results

summary(results)

# Plot the weights of the mixture components

install.packages("ggplot2") # Install ggplot2 for visualization

library(ggplot2)

# Sort the weights

w_ord <- order(results$final_weights$mean_weight)

mean_weight <- results$final_weights$mean_weight[w_ord]

mix_name <- factor(results$final_weights$mix_name[w_ord], levels = results$final_weights$mix_name[w_ord])

# Prepare data for visualization

data_plot <- data.frame(mean_weight, mix_name)

# Create the bar plot of weights

ggplot(data_plot, aes(x = mix_name, y = mean_weight, fill = mix_name)) +

geom_bar(stat = "identity", color = "black") + theme_bw() +

theme(axis.ticks = element_blank(),

axis.title = element_blank(),

axis.text.x = element_text(color = 'black'),

legend.position = "none") + coord_flip()

# Display the detailed weight values

options(digits = 3) # Set the number format to decimal instead of scientific notation

gwqs_weights_tab(results) # Display detailed weight values

# Overall effect summary

gwqs_summary_tab(results) # Summary table of overall effects

# Scatterplot of the results

gwqs_scatterplot(results) # Fitting plot

# Run the inverse direction of the data

results <- gwqs(sarcopenia ~ wqs + age + race + gender + education_level +

marital_status + BMI + smoking_status + alcohol_drinking + hypertension +stroke + diabetes + WBC + ALP + platelet + vitamin_D + triglycerides + HDL_C,

mix_name = toxic_chems, # Specify the mixture dataset

data = a, q = 4, validation = 0.6, b = 100, # Original dataset and number of bootstrap samples

b1_pos = FALSE, b1_constr = FALSE, family = "binomial", # Define direction and type of outcome variable

seed = 2000, plots = TRUE, tables = TRUE) # Set seed for reproducibility and enable plots/tables

1. **Bayesian kernel machine regression analysis (BKMR) R Code:**

# Set working directory

setwd("C:/Users/lenovo/Desktop")

# Install and load necessary packages

library(data.table)

install.packages("bkmr")

install.packages("readxl")

install.packages("corrplot")

library(bkmr)

library(corrplot)

library("ggplot2")

# Load the dataset

a <- fread("data.csv") # It is important to load all environmental exposure data together

# View dataset variables

names(a)

# Define exposure variables (from column 63 to 77)

mixture <- as.matrix(a[, 63:77])

# Define covariates (from columns 7-8, 10-20, and 26)

covariates <- as.matrix(a[, c(7:8, 10:20, 26)])

# Define the outcome variable

y <- a$anxiety

# Perform Bayesian Kernel Machine Regression (BKMR)

knots50 <- fields::cover.design(mixture, nd = 50)$design

fit2 <- kmbayes(y = y, Z = mixture, X = covariates, iter = 5000, verbose = FALSE, varsel = TRUE, knots = knots50)

# Extract Posterior Inclusion Probabilities (PIP)

ExtractPIPs(fit2)

# Univariate exposure-response plot

pred.resp.univar <- PredictorResponseUnivar(fit = fit2)

ggplot(pred.resp.univar, aes(z, est, ymin = est - 1.96 * se, ymax = est + 1.96 * se)) +

geom_smooth(stat = "identity") + facet_wrap(~ variable) + ylab("h(z)") # Visualization

# Overall risk summary for mixture effects

risks.overall <- OverallRiskSummaries(fit = fit2, qs = seq(0.25, 0.75, by = 0.05), q.fixed = 0.5)

ggplot(risks.overall, aes(quantile, est, ymin = est - 1.96 * sd, ymax = est + 1.96 * sd)) + geom_pointrange() + geom_hline(yintercept = 0, lty = 2, col = "red")

# Single-variable risk summary

risks.singvar <- SingVarRiskSummaries(fit = fit2, y = y, Z = mixture, X = covariates, qs.diff = c(0.25, 0.75), q.fixed = c(0.25, 0.50, 0.75))

ggplot(risks.singvar, aes(variable, est, ymin = est - 1.96 * sd, ymax = est + 1.96 * sd, col = q.fixed)) +

geom_pointrange(position = position_dodge(width = 0.75)) + geom_hline(yintercept = 0, lty = 2, col = "red")

# Plot interaction effects between exposure variables

expos.pairs <- subset(data.frame(expand.grid(expos1 = c(1:15), expos2 = c(1:15))), expos1 < expos2)

pred.resp.bivar <- PredictorResponseBivar(fit = fit2, min.plot.dist = 1, z.pairs = expos.pairs)

pred.resp.bivar.levels <- PredictorResponseBivarLevels(pred.resp.bivar, mixture, qs = c(0.10, 0.5, 0.90))

ggplot(pred.resp.bivar.levels, aes(z1, est)) + geom_smooth(aes(col = quantile), stat = "identity") + facet_grid(variable2 ~ variable1) + ggtitle("h(expos1 | quantiles of expos2)") + xlab("expos1")

# Group-wise Posterior Inclusion Probabilities (GroupPIP)

hier <- kmbayes(y = y, Z = mixture, X = covariates, iter = 200, verbose = FALSE, varsel = TRUE, groups = c(1,1,2,2,3,3,2,2,4,4,2,4,2,4,2))

ExtractPIPs(hier)
